# The most effective combination of pharmacological therapy for heart failure with reduced ejection fraction: a network meta-analysis of randomized controlled trials

**DOI:** 10.1186/s12872-024-04339-3

**Published:** 2024-11-23

**Authors:** Huilin Tang, Kimberly Germinal, Alexandra Milfort, Wei-Han Chen, Shao-Hsuan Chang, Wenxi Huang, Yujia Li, Ying Lu, Mustafa M. Ahmed, Stephen E. Kimmel, Jiang Bian, Jingchuan Guo

**Affiliations:** 1 Department of Pharmaceutical Outcomes and Policy, University of Florida College of Pharmacy, Gainesville, FL USA; 2 Division of Cardiovascular Medicine, College of Medicine, University of Florida, Gainesville, FL USA; 3 Department of Epidemiology, College of Public Health and Health Professions and College of Medicine, University of Florida, Gainesville, FL USA; 4 Department of Health Outcomes and Biomedical Informatics, College of Medicine, University of Florida, Gainesville, FL USA; 5 Center for Drug Evaluation and Safety, University of Florida, Gainesville, FL USA

**Keywords:** Heart failure with reduced ejection fraction, Pharmacological interventions, Randomized controlled trials, Meta-analysis

## Abstract

**Background:**

Evidence for the efficacy of pharmacological therapies for heart failure with reduced ejection fraction (HFrEF) is growing. However, there is no consensus on the most effective treatment for HFrEF. This study aimed to evaluate the most effective combination of pharmacological therapy in patients with HFrEF.

**Methods:**

We systematically searched Medline, Embase, and CENTRAL up to Feb 2022, to include randomized controlled trials (RCTs) that evaluated the efficacy of pharmacological treatment among adults (≥ 18 years) with a diagnosis of HFrEF (defined by a left ventricular ejection fraction ≤ 45%). The outcomes of interest included all-cause death, cardiovascular (CV) death, and hospitalization for heart failure (HHF). A random network meta-analysis using a frequentist framework model was employed to calculate the pooled risk ratio (RR) with 95% confidence interval (CI) and rank the treatments.

**Results:**

We included 49 RCTs involving 90,529 participants with HFrEF. For reducing all-cause mortality, the combination of angiotensin-converting enzyme inhibitors (ACEI), beta-blockers (BB), mineralocorticoid receptor antagonists (MRA), and sodium-glucose co-transporter-2 inhibitors (SGLT2i) was most effective (RR, 0.46; 95% CI, 0.32–0.66). For CV death, the combination of ACEI, BB, MRA, and Vericiguat showed the highest efficacy (RR, 0.34; 95% CI, 0.12–0.90). Regarding reducing HHF, the combination of ACEI, BB, MRA, and SGLT2i as well as the combination of ACEI, BB, MRA, and Ivabradine were equally the most effective (both RR, 0.27; 95% CI, 0.18–0.39).

**Conclusion:**

This study provides robust evidence supporting the use of combination therapies in HFrEF management, with newer agents offering incremental benefits when added to established guideline-directed medical therapy.

**Supplementary Information:**

The online version contains supplementary material available at 10.1186/s12872-024-04339-3.

## Introduction

Heart failure (HF) is a chronic and progressive disease with complex and variable symptoms characterized by dyspnea or exertional limitation [1]. It affects about 26 million people worldwide [2], of which, approximately 50% are HF with reduced ejection fraction (HFrEF) [1, 3]. HFrEF is characterized by a left ventricular ejection fraction (LVEF) of less than 40% as a result of chronic cardiac remodeling causing unwanted dilation of the left ventricle [1]. The underlying cause of HFrEF tends to be coronary artery disease with many patients living with the burden of comorbidities such as hypertension, chronic kidney disease, and diabetes [3]. The expansion of HFrEF research remains vital as recent studies show that 5-year mortality rates of these patients are between 45–60% [4]. Despite the advances in treatment and prevention strategies, HFrEF remains a major public health concern with substantial morbidity and mortality, making it an ongoing issue that requires attention and resources for improvement [3].

Management of HFrEF has undergone a significant evolution in recent years.

More recent developments including sodium-glucose co-transporter-2 inhibitors (SGLT2i), ivabradine, and Hydralazine and Isosorbide Dinitrate (H-ISDN) have significantly advanced clinical practices by decreasing the morbidity and mortality in high-risk patients with HFrEF [5]. Focusing on SGLT2i, while type 2 diabetes is regarded as a risk factor for the development of heart failure, this class of medications has been found to be beneficial regardless of diabetes status [6]. Clinical trials such as DAPA-HF and EMPEROR-Reduced found the risk of hospitalization was decreased by more than 30% and the risk of cardiovascular (CV) death was decreased by 14% utilizing SGLT2i marking their importance in the management of HFrEF [7, 8]. Angiotensin II converting enzyme inhibitors (ACEI), angiotensin II receptor blockers (ARB), or angiotensin II receptor-neprilysin inhibitors (ARNI) remain recommended by AHA/ACC/HFSA guidelines and CCS/CHFS guidelines as first-line therapy for patients with HFrEF to reduce morbidity and mortality [5, 9]. ARNI medications have become preferred over the traditional ACEI and ARB treatment options as clinical trials such as PARADIGM-HF have proven that ARNI was able to reduce CV death by greater than 20% and hospitalization by 12% in comparison to ACEI [10]. Other drug classes including beta-blockers (BB), mineralocorticoid receptor antagonists (MRA), and soluble guanylate cyclase have been recommended for the resolution of symptom treatment in HFrEF patients [5]. Medication recommendations are made based on comorbidities such as decreased renal function, presence of symptoms, and tolerance to previous treatment [5]. With these varying criteria, there is a lack of uniformity between HFrEF patients making individual medication efficacy more difficult to predict.

Despite established evidence that current pharmacological therapies reduce morbidity and mortality in HFrEF, optimizing HFrEF treatment regimens remains challenging due to the complex pathophysiology of HFrEF. Therefore, we performed a network meta-analysis (NMA) of randomized controlled trials (RCTs) to identify the most optimal combination of pharmacological therapy in patients with HFrEF. This was assessed by the largest p-score in the outcome of all-cause death, CV death, and hospitalization for heart failure (HHF).

## Methods

This network meta-analysis was conducted according to the PRISMA extension statement for the reporting of systematic reviews incorporating network meta-analyses of healthcare interventions [11].

### Search strategy and study selection

We comprehensively searched PubMed, Embase, and Cochrane Central Register of Controlled Trials (CENTRAL) from inception to Feb 9, 2022 to identify eligible RCTs. A detailed search strategy that included electronic databases and key terms is presented in File S1. In addition, we also identified other potential trials by manually searching the reference lists of included trials and relevant meta-analyses.

Three reviewers (WH, WC, and SC) selected the trials based on the following inclusion criteria: 1) RCTs that included adults (aged ≥ 18 years) with a diagnosis of HFrEF with a definition of LVEF ≤ 45%; 2) trial comparing one pharmacological treatment with another pharmacological treatment or placebo/no use; and 3) trials reporting at least one of the following outcomes including all-cause death, CV death, and HHF; 4) Trials with a sample size ≥ 100. The drugs of interest included ACEI, ARB, ARNI, BB, MRA, SGLT2i, Ivabradine, Omecamtiv Mecarbil, and vericiguat. In the trials, the use of these other drugs was considered as background therapy if 60% of participants took these drugs. Outcomes of interest were all-cause death, CV death, and HHF.

### Data extraction and quality assessment

Four reviewers (WH, WC, SC, and YL) extracted data from original trial reports using a standardized form. Two reviewers were assigned in a group and cross-checked the data. Data extracted included study characteristics (first author, publication year, NCT number, and duration of follow-up) and characteristics of patients (inclusion criteria, background treatments, mean age, and proportion of men), treatments of interest, comparators, and the outcomes of interest. If multiple reports from the same population were retrieved, only the most complete and/or most recently reported data were used.

Similarly, two reviewers in a group assessed the study quality using the Cochrane risk of bias tool as described in the Cochrane Collaboration Handbook [12]. We assessed the risk of bias in the following 6 domains: random sequence generation, allocation concealment, blinding (participants and personnel), blinding (outcome assessment), incomplete outcome data (attrition bias), and selective reporting (reporting bias). The risk of bias for each domain was judged as low risk, high risk, or unclear risk. The overall risk of bias of the trial was classified as high for the one with at least one domain judged as high and unclear for the one with a least one domain judged as unclear.

### Statistical analysis

We conducted a frequentist-based NMA using graph-theoretical methods developed for electrical networks [13, 14]. NMA was chosen for its ability to comprehensively compare multiple treatments simultaneously, utilize both direct and indirect evidence, rank treatments based on efficacy, and increase statistical power by combining data from multiple trials [15]. This approach is particularly valuable in the complex landscape of HFrEF treatments, where numerous pharmacological options exist and direct comparisons between certain treatments are limited. To account for the potential heterogeneity between studies, we applied random effects models to calculate the risk ratio (RR) and 95% confidence interval (CI) for all-cause death, CV death, and HHF. First, we evaluated the above outcomes for available treatments within the network compared with a placebo using standard NMA. Second, we applied an additive component NMA (CNMA) model to evaluate individual component effects on the risk of outcomes due to the complexation of pharmaceutical treatments for HFrEF that consist of multiple components [16]. The additive CNMA model assumes that the effects of individual components are additive, meaning that the total effect of treatment combinations is the sum of the effects of its components [16, 17]. The advantages of additive CNMA include 1) it breaks down the intervention (e.g., ARNI + BB + MRA) into their constituent components (ARNI vs. BB vs. MRA) instead of treating each intervention as a single unit; 2) it allows to estimate the effect of interventions that have not been directly compared in trials by combing information on their components; 3) it provides more precise estimates than standard NMA, especially direct evidence is limited; 4) it can help identify which components of complex interventions are most effective. We also assessed the relative ranking of treatments using their surface under the cumulative ranking curve (SUCRA) score (also known as the P-score), which represents their likelihood of being ranked the most effective. In this study, larger P-score indicates a lower risk [18]. A sensitivity analysis was performed among participants with LVEF ≤ 40%.

To check the consistency assumption within the network model, we compared the consistency between direct and indirect evidence for each comparison using the node-splitting method [19, 20]. If there is significant disagreement (p < 0.05), it suggests inconsistency. We also applied I^2^ statistic to measure the heterogeneity (the degree of disagreement between study-specific treatment effects) [21]. I^2^ statistic and the 95% CI were interpreted as 0–25%, 25- 75%, and > 75%, indicating low, moderate, and high heterogeneity, respectively. Finally, a comparison-adjusted funnel plot and Egger’s test were used to assess the potential publication bias and small study effects within a network of interventions [22].

The analysis was performed using the “netmeta” package in R (R Foundation). A two-tailed P < 0.05 was considered statistically significant.

## Results

### Study selection and Study characteristics

**Fig. 1:**
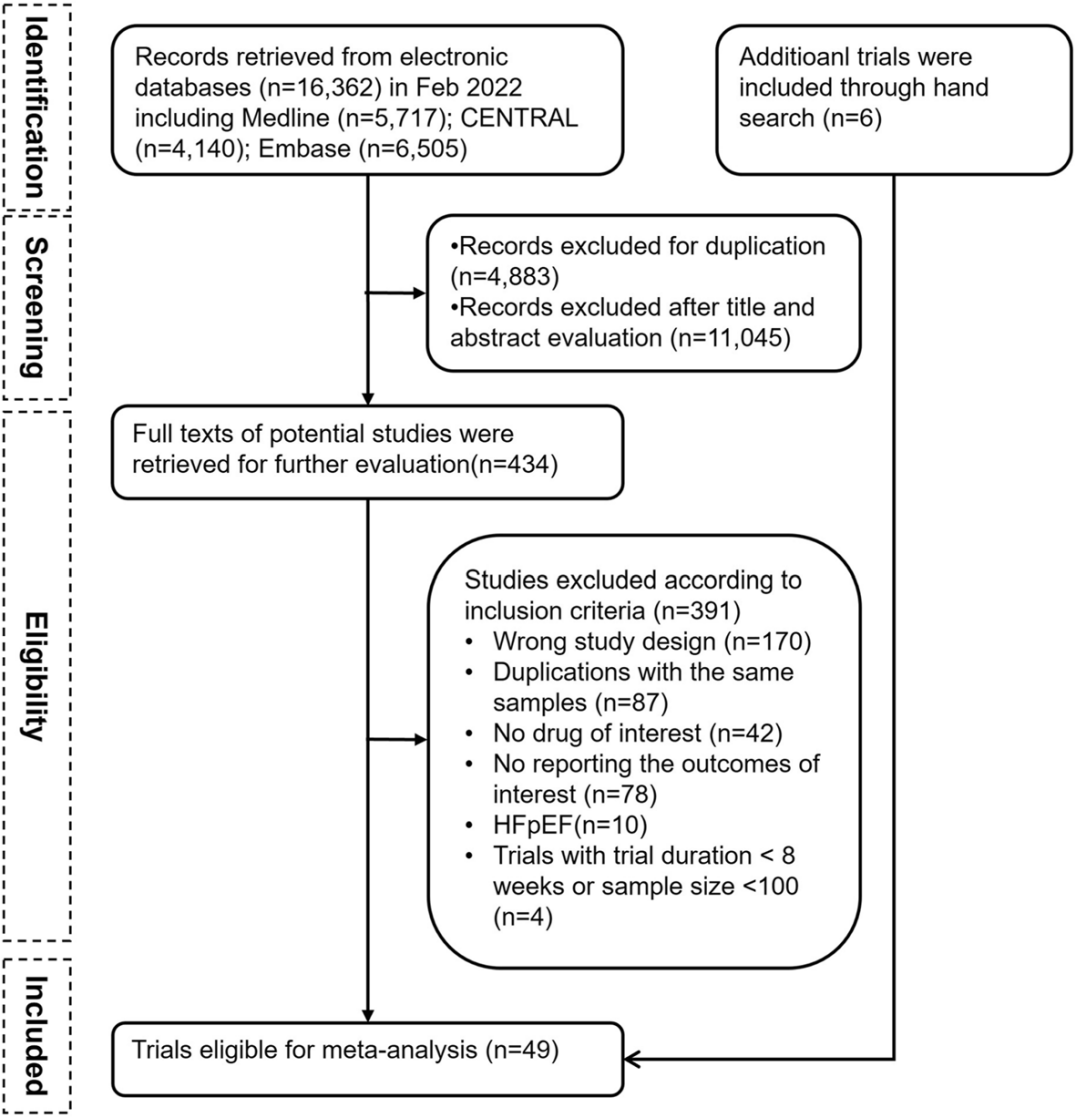
The flowchart of trial selection. HFpEF, heart failure with preserved ejection fraction; CENTRAL, Cochrane Central Register of Controlled Trials

**Fig. 2:**
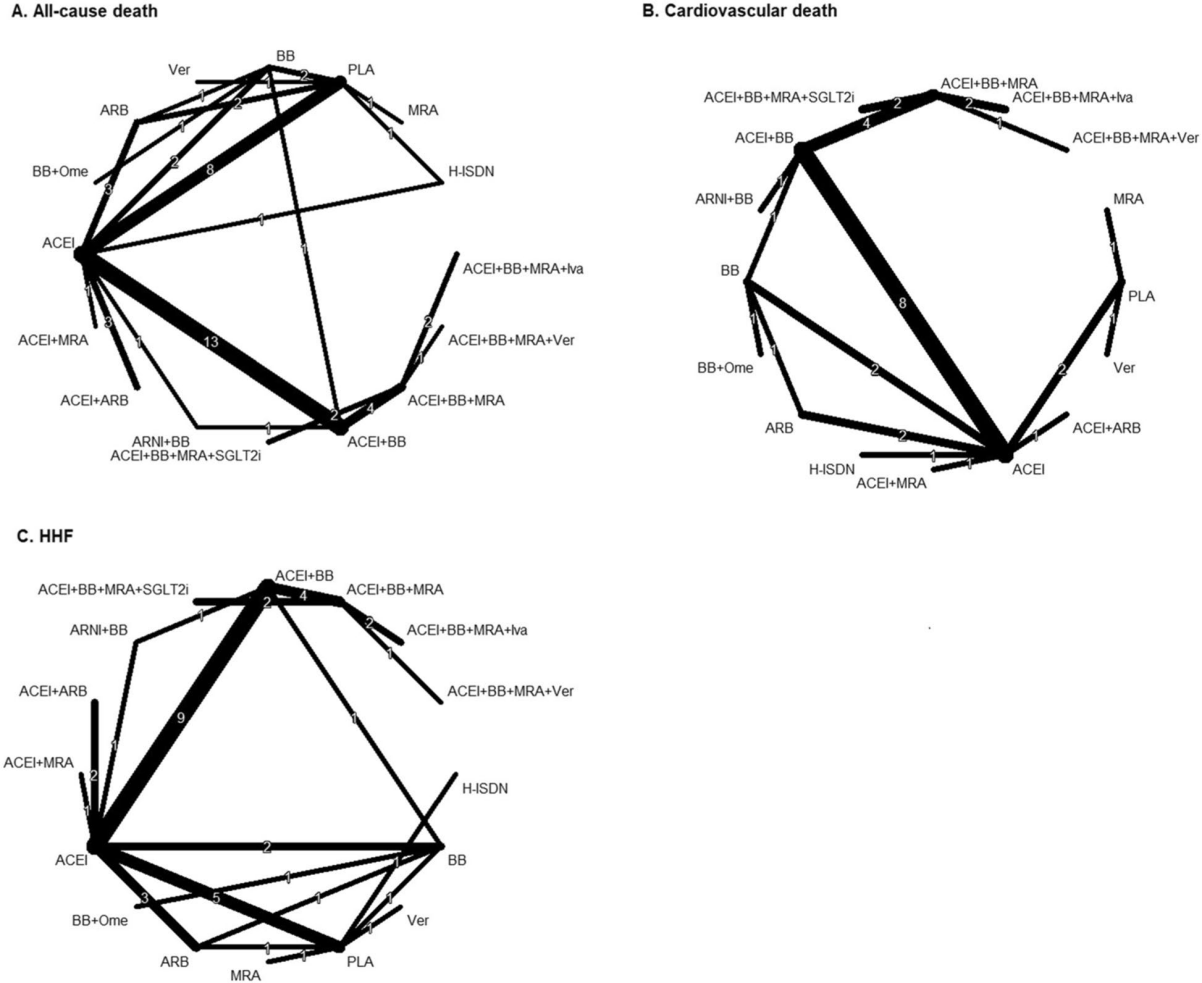
Network graphs of pharmacological treatments for heart failure with reduced ejection fraction in terms of all-cause death(**A**), cardiovascular death (**B**), and hospitalization for heart failure (HHF) (**C**). SGLT2i, sodium-glucose co-transporter-2 inhibitors; H-ISDN, Hydralazine and Isosorbide Dinitrate; ACEI, angiotensin II converting enzyme inhibitors; ARB, angiotensin II receptor blockers; ARNI, angiotensin II receptor-neprilysin inhibitors; BB, beta blocker; MRA, mineralocorticoid receptor antagonists; Iva, Ivabradine; Ver, Vericiguat; Ome, Omecamtiv

Figure 1 shows the process of identifying eligible trials. A total of 16,362 citations were retrieved through our electronic search. After screening the titles/abstracts and full texts based on the inclusion and exclusion criteria, we included 44 studies. Along with the 6 trials identified through manual search, 49 trials were finally included [7, 8, 10, 23–68]. The clinical characteristics of each study are presented in Table 1. A total of 90,529 participants with HFrEF from 49 independent trials were included. The mean sample size for each trial was 1,847 (range: 100 – 8,442), and the mean duration of follow-up was 17.2 months (range: 2—71.8 months). Participants were generally middle-aged (mean age: 61.8 years), with a percentage of men at 77.5%. The available direct comparisons and network of trials are shown in Fig. 2.

**Table 1:** Baseline characteristics of included randomized controlled trials

Study	Trial name	Population definition	No	Age, years	Male, %	Background Therapy (≥ 60%)	Treatment	Control	follow-up, months
**Armstrong 2020** [28]	VICTORIA	Patients with NYHA class II-IV HF and EF < 45%	5050	67.3	76	-	Vericiguat	Placebo	10.8
**Beller 1995** [29]	-	Patients with NYHA class II-IV and LVEF ≤ 45%	193	59.6	75.1	-	ACEI	Placebo	3
**BEST 2001** [30]	BEST	Patients with NYHA class III or IV HF and LVEF ≤ 35%	2708	60	78	ACEI	BB	Placebo	24
**Boccanelli 2009** [31]	AREA IN-CHF	Patients with NYHA class II HF and LVEF < 45%	467	62.5	83.5	-	MRA	Placebo	12
**Bristow 1996** [32]	MOCHA	Patients with HF symptoms and EF ≤ 35%	345	60	76	ACEI	BB	Placebo	6
**Brown 1995** [33]	-	Patients with NYHA class II or III HF and LVEF ≤ 35%	241	62	79.7	-	ACEI	Placebo	6
**Captopril-Digoxin group 1988** [23]	-	Patients with LVEF ≤ 40%	300	56.8	83	-	ACEI	Placebo	6
**CIBIS I 1994** [24]	CIBIS I	Patients with chronic HF and LVEF < 40%	641	60	83	ACEI	BB	Placebo	22.8
**CIBIS-II 1999** [25]	CIBIS-II	Patients with NYHA class III or IV HF and LVEF ≤ 35%	2647	61	80	ACEI	BB	Placebo	15.6
**Cohn 1997** [34]	-	Patients with HF symptoms and LVEF ≤ 35%	105	60	58	ACEI	BB	Placebo	NR
**Cohn 2001** [35]	Val-HeFT	Patients with NYHA class II-IV HF and LVEF ≤ 40%	5010	62.7	80	ACEI	ARB	Placebo	23
**Colucci 1996** [36]	-	Patients with CHF and LVEF ≤ 35%	366	55	85	-	BB	Placebo	7
**Dargie 2001** [37]	CAPRICORN	Patients with proven acute myocardial infarction and an LVEF ≤ 40%	1959	63	70	ACEI	BB	Placebo	15.6
**Dunselman 2001** [38]	REPLACE	Patients with chronic HF and LVEF ≤ 40%	378	64	89	ACEI	ARB	ACEI	3
**Erhardt 1995** [39]	FEST	Patients with NYHA class II or III HF and LVEF ≤ 35%	308	63	74	-	ACEI	Placebo	3
**Fonarow 1992** [40]	Hy-C Trial	Patients with severe HF and mean LVEF = 20%	117	51.8	90	-	ACEI	H-ISDN	8
**Gheorghiade 2015** [41]	SOCRATES reduced	Patients with HF and LVEF < 45%	456	68	80.3	ACEI + BB + MRA	Vericiguat	Placebo	4
**Granger 2000** [42]	SPICE	Patients with NYHA class Il—IV HF and LVEF ≤ 35%	270	65.7	70	-	ARB	Placebo	3
**He 2015** [43]	NA	Patients with NYHA class II–IV HF and LVEF ≤ 35%	491	50	60	-	ACEI	BB	50.4
**Hori 2004** [44]	MUCHA	Patients with NYHA class II or III and LVEF ≤ 40%	174	60	78	ACEI	BB	Placebo	Up to 12
**Komajda 2004** [47]	CARMEN	Patients with stable mild CHF and LVEF < 40%	572	62.3	80.7	ACEI	ACEI + BB	BB	18
**McMurray 2003** [48]	CHARM-Added	Patients with NYHA class II–IV HF and LVEF ≤ 40%	2548	64	80	ACEI	ARB	Placebo	41
**McMurray 2014** [10]	PARADIGM-HF	Patients with NYHA class II—IV symptoms and LVEF ≤ 40%	8442	63.8	80	BB	ARNI	ACEI	27
**McMurray 2019** [7]	DAPA-HF	Patients with NYHA class II-IV HF and LVEF ≤ 40%	4744	66.4	76.6	ACEI* + BB + MRA	SGLT2i	Placebo	18
**MERIT-HF** [26]	MERIT-HF	Patients with symptomatic HF and an ejection fraction of ≤ 40%	3991	-	77.5	ACEI	BB	Placebo	12
**Packer 1996a** [49]	-	Patients with HF symptoms and EF ≤ 35%	1094	58	76.6	ACEI	BB	Placebo	6.5
**Packer 1996b** [51]	PRECISE	Patients with moderate to severe HF and LVEF ≤ 35%	278	60.3	73.4	ACEI	BB	Placebo	6
**Packer 2001** [50]	COPERNICUS	Patients with severe CHF and LVEF < 25%	2289	63.3	79.5	ACEI	BB	Placebo	10.4
**Packer 2020** [8]	EMPEROR-Reduced	Patients with CHF and LVEF ≤ 40%	3730	66.8	76.1	ACEI* + BB + MRA	SGLT2i	Placebo	16
**Pitt 1997** [54]	ELITE	Patients with NYHA II–IV HF and LVEF ≤ 40%	722	73.5	70		ARB	ACEI	11
**Pitt 1999** [55]	RALES	Patients with severe HF and LVEF ≤ 35%	1663	65	73	ACEI	MRA	Placebo	24
**Pitt 2000** [52]	ELITE II	Patients with NYHA class II-IV HF and LVEF ≤ 40%	3152	71.4	70		ARB	ACEI	18.5
**Pitt 2003** [53]	EPHESUS	Patients with HF and LVEF ≤ 40%	6200	64	70	ACEI* + BB	MRA	Placebo	16
**RESOLVD 2000** [27]	RESOLVD (pilot)	Patients with NYHA class II to IV CHF and LVEF < 40%	426	62	82	-	BB	Placebo	6
**Riegger 1999** [56]	STRETCH	Patients with congestive HF and LVEF (30% to 45%)	844	62	68.4	-	ARB	Placebo	3
**SOLVD-prevent 1992** [46]	SOLVD-prevent	Patients with HF and EF ≤ 35%	4228	59.1	88.5	-	ACEI	Placebo	37.4
**SOLVD-treat 1991** [45]	SOLVD-treat	Patients with congestive HF and EF ≤ 35%	2569	60.9	80.4	-	ACEI	Placebo	41.4
**Sturm 2000** [57]	-	Patients with HF and LVEF ≤ 25%	100	51.5	88	ACEI	BB	Placebo	32.9
**Swedberg 2010** [58]	SHIFT	Patients with symptomatic HF and LVEF ≤ 35%	6558	60.4	76	ACEI + BB + MRA	Ivabradine	Placebo	22.9
**Taylor 2004** [59]	A-HeFT	Patients with NYHA class III or IV HF and LVEF < 45%	1050	56.8	59.9	-	H-ISDN	Placebo	18
**Teerlink 2021** [60]	GALACTIC-HF	Patients with symptomatic chronic HF and EF ≤ 35%	8256	64.5	78.8	ACEI* + BB + MRA	Omecamtiv	Placebo	21.8
**Tsutsui 2017** [61]	J-EMPHASIS-HF	Patients with NYHA class II–IV and an EF ≤ 35%	221	68.7	88	ACEI + BB	MRA	Placebo	71.8
**Tsutsui 2019** [62]	J-SHIFT	Patients with stable symptomatic chronic HF and LVEF ≤ 35%	254	60.7	82.3	ACEI + BB + MRA	Ivabradine	Placebo	19.6
**Velazquez 2019** [64]	PIONEER-HF	Patients with symptoms of HF and LVEF ≤ 40%	881	62	70	BB	ARNI	ACEI	2
**Veldhuisen 1998** [63]	-	Patients with mild to moderate CHF and LVEF < 45%	244	61	77	-	ACEI	Placebo	3
**Vizzardi 2014** [65]	-	Patients with NYHA classes I—II HF and LVEF < 40%	130	62	-	ACEI* + BB	MRA	Placebo	44
**Widimský 1995** [66]	CASSIS	Patients with CHF and either LVEF < 40%	248	57.5	83	-	ACEI	Placebo	3
**Witchitz 2000** [67]	CELICARD	Patients with NYHA class II to IV and LVEF < 40%	132	56.5	89.5	ACEI	BB	Placebo	12
**Zannad 2011** [68]	EMPHASIS-HF	Patients with NYHA class II HF and LVEF ≤ 35%	2737	68.7	77.7	ACEI + BB	MRA	Placebo	21

^*^Renin–Angiotensin–Aldosterone System (RAAS) inhibitors while ACEI accounts for the most

HF, chronic heart failure; LVEF, left ventricular ejection fraction; NYHA, New York Heart Association, SGLT2i, sodium-glucose co-transporter-2 inhibitors, H-ISDN, Hydralazine and Isosorbide Dinitrate, ACEI, angiotensin II converting enzyme inhibitors, ARB, angiotensin II receptor blockers, ARNI, angiotensin II receptor-neprilysin inhibitors, BB, beta blocker, *MRA* mineralocorticoid receptor antagonists, *NR* not reported

### Risk of *bias* and publication *bias*

The risk of bias assessment for the 49 trials included in this meta-analysis generally showed a low to unclear risk across most domains (Table S1). Random sequence generation and allocation concealment were the domains most frequently rated as unclear, particularly in earlier studies published before 2000. Blinding of participants, personnel, and outcome assessment was predominantly low risk across trials. Selective reporting and incomplete outcome data were also generally low risk. More recent trials, especially those involving newer drugs like SGLT2i, tended to have low risk of bias across all domains. Overall, the majority of trials were assessed as having low overall risk of bias except for some earlier studies. In addition, the comparison-adjusted funnel plot and Egger’s test indicated the absence of publication bias or small-study effects (Figure S1).

### All-cause death

**Fig. 3:**
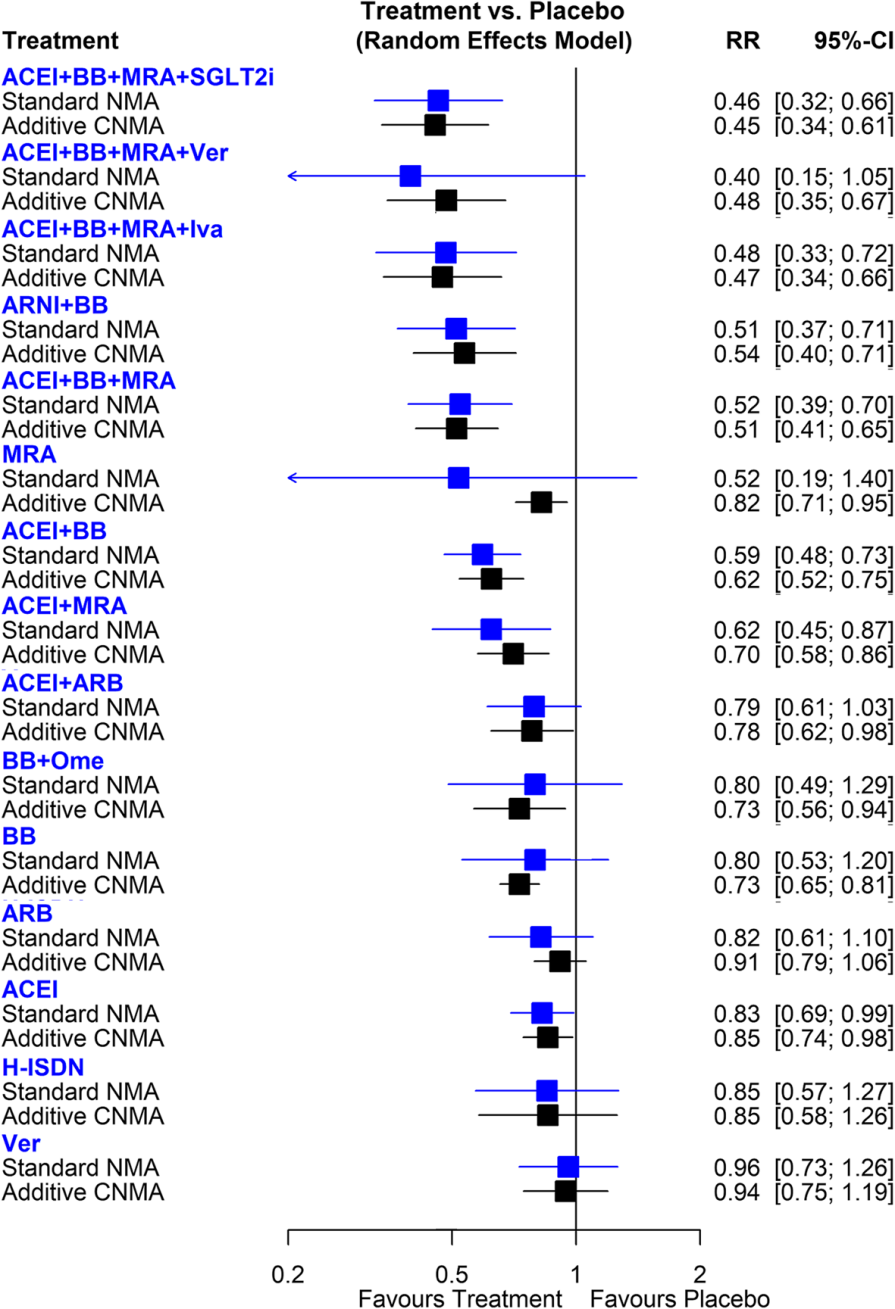
Forest plots of frequentist standard network meta-analysis (NMA) and additive component NMA (CNMA) of pharmaceutical treatments for risk of all-cause death in patients with HFrEF. RR, risk ratio; CI, confidence interval; SGLT2i, sodium-glucose co-transporter-2 inhibitors; H-ISDN, Hydralazine and Isosorbide Dinitrate; ACEI, angiotensin II converting enzyme inhibitors; ARB, angiotensin II receptor blockers; ARNI, angiotensin II receptor-n eprilysin inhibitors; BB, beta blocker; MRA, mineralocorticoid receptor antagonists; Iva, Ivabradine; Ver, Vericiguat; Ome, Omecamtiv

Figure 2 shows the network of eligible comparisons for all-cause death, involving 48 RCTs. The results for all-cause death in standard NMA are presented in Fig. 3 and Table S2. Compared with placebo, ACEI + BB + MRA + SGLT2i (RR, 0.46; 95%CI, 0.32 – 0.66), ACEI + BB + MRA + Ivabradine (RR, 0.48; 95%CI, 0.33–0.72), ARNI + BB (RR, 0.51; 95%CI, 0.37–0.71), ACEI + BB + MRA (RR, 0.52; 95%CI, 0.39–0.70), ACEI + BB (RR, 0.59; 95%CI, 0.48–0.73), and ACEI + MRA (RR, 0.62; 95%CI, 0.45–0.87) were significantly associated with a lower risk of all-cause death. The p-score showing the overall ranking of each treatment indicated that ACEI + BB + MRA + SGLT2i was ranked as the best approach (p-score = 0.86), followed by ACEI + BB + MRA + Vericiguat (p-score = 0.83), and ACEI + BB + MRA + Ivabradine (p-score = 0.82) (Table S5). The point estimates were similar in additive CNMA (Fig. 3). Because of the increased number of available treatment arms and potential interaction effects in the combination therapies in additive CNMA, several interventions had a narrower CI, resulting in a significant effect, such as ACEI + BB + MRA + Vericiguat (RR, 0.48; 95%CI, 0.35–0.67), BB + Omecamtiv (RR, 0.73; 95%CI, 0.56–0.94), BB (RR, 0.73; 95%CI, 0.65–0.81), and MRA (RR, 0.82; 95%CI, 0.71–0.95). The effects of individual components on all-cause death risk are present in Table S6. ARNI, ACEI, BB, and MRA were associated with a lower risk of all-cause death.

When checking the consistency in the network, direct and indirect drug comparisons were generally consistent except for the comparison of ACEI vs H-ISDN (p = 0.02). The heterogeneity was moderate in the network meta-analysis, with I^2^ = 43.2% (95%CI, 15.5%- 61.8%).

### Cardiovascular death

**Fig. 4:**
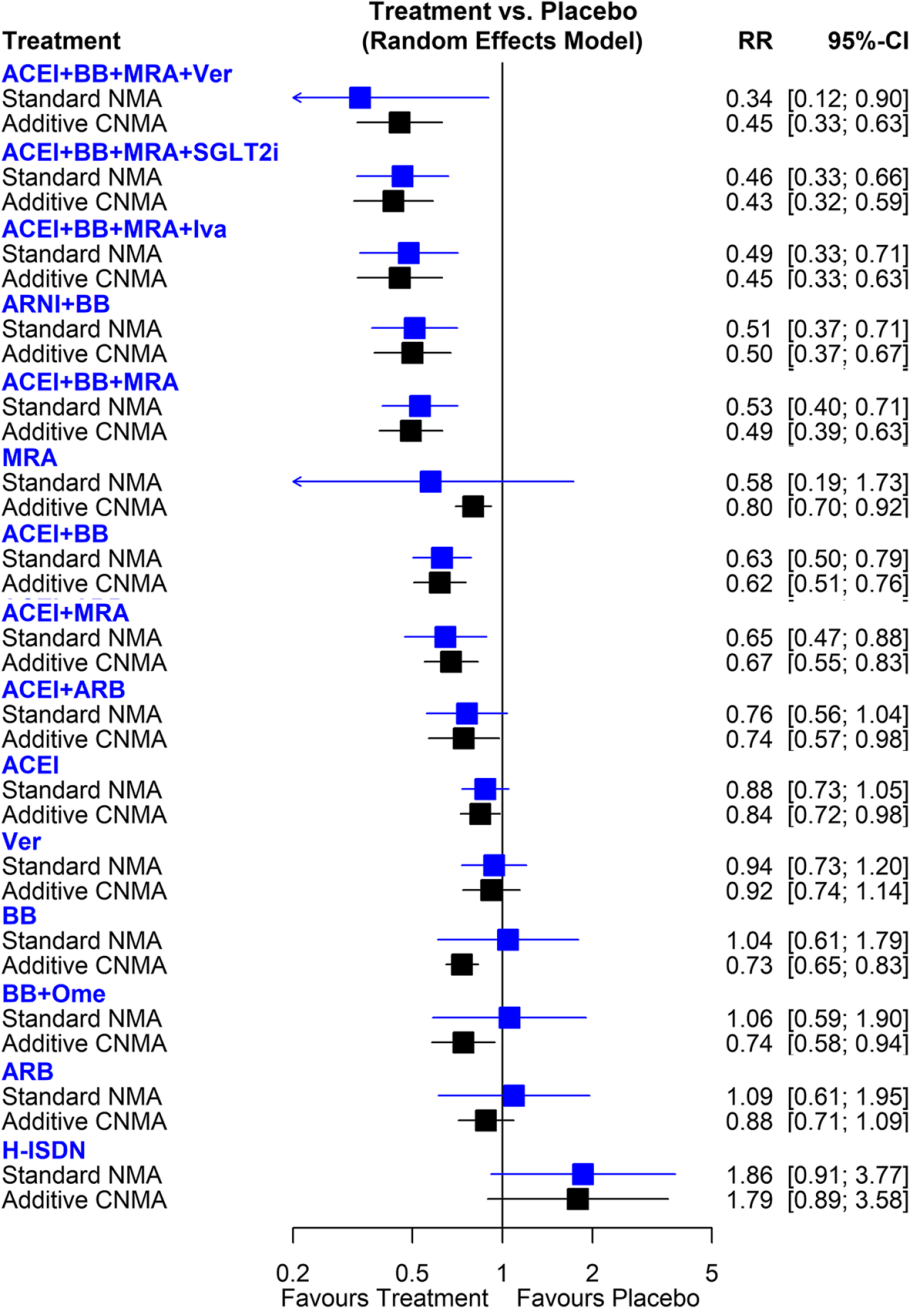
Forest plots of frequentist standard network meta-analysis (NMA) and additive component NMA (CNMA) of pharmaceutical treatments for risk of cardiovascular death in patients with HFrEF. RR, risk ratio; CI, confidence interval; SGLT2i, sodium-glucose co-transporter-2 inhibitors; H-ISDN, Hydralazine and Isosorbide Dinitrate; ACEI, angiotensin II converting enzyme inhibitors; ARB, angiotensin II receptor blockers; ARNI, angiotensin II receptor-neprilysin inhibitors; BB, beta blocker; MRA, mineralocorticoid receptor antagonists; Iva, Ivabradine; Ver, Vericiguat; Ome, Omecamtiv

Figure 2 shows the network of eligible comparisons for CV death, involving 28 RCTs. The standard NMA results for CV death are presented in Fig. 4 and Table S3. Compared with placebo, ACEI + BB + MRA + Vericiguat (RR, 0.34; 95%CI, 0.12—0.90), ACEI + BB + MRA + SGLT2i (RR, 0.46; 95%CI, 0.33–0.66), ACEI + BB + MRA + Ivabradine (RR, 0.49; 95%CI, 0.33–0.71), ARNI + BB (RR, 0.51; 95%CI, 0.37–0.71), ACEI + BB + MRA (RR, 0.53; 95%CI, 0.40–0.71), ACEI + BB (RR,0.63; 95%CI, 0.50–0.79), and ACEI + MRA (RR,0.65; 95%CI, 0.47–0.88) were significantly associated with a lower risk of CV death. The p-score of an overall ranking of each treatment indicated that ACEI + BB + MRA + Vericiguat was ranked as the best approach (p-score = 0.90), followed by ACEI + BB + MRA + SGLT2i (p-score = 0.87), and ACEI + BB + MRA + Ivabradine (p-score = 0.83) (Table S5). The point estimates were similar for most comparisons in both standard NMA and additive CNMA (Fig. 4). Because of the increased number of available treatment arms and potential interaction effects in the combination therapies in additive CNMA, several interventions had a narrower CI, resulting in a significant effect, such as ACEI + ARB (RR, 0.74; 95%CI, 0.57–0.98), ACEI (RR, 0.84; 95%CI, 0.72–0.98), BB + Omecamtiv (RR, 0.74; 95%CI, 0.58–0.94), BB (RR, 0.73; 95%CI, 0.65–0.83) and MRA (RR, 0.80; 95%CI, 0.70–0.92). The effects of individual components on all-cause death risk are present in Table S6. ARNI, ACEI, BB, and MRA were associated with a lower risk of all-cause death.

When checking the consistency in the network, direct and indirect drug comparisons were generally consistent. However, there was moderate heterogeneity in the network meta-analysis, with I^2^ = 41.2% (0.0%; 67.5%).

### Hospitalization for heart failure

**Fig. 5:**
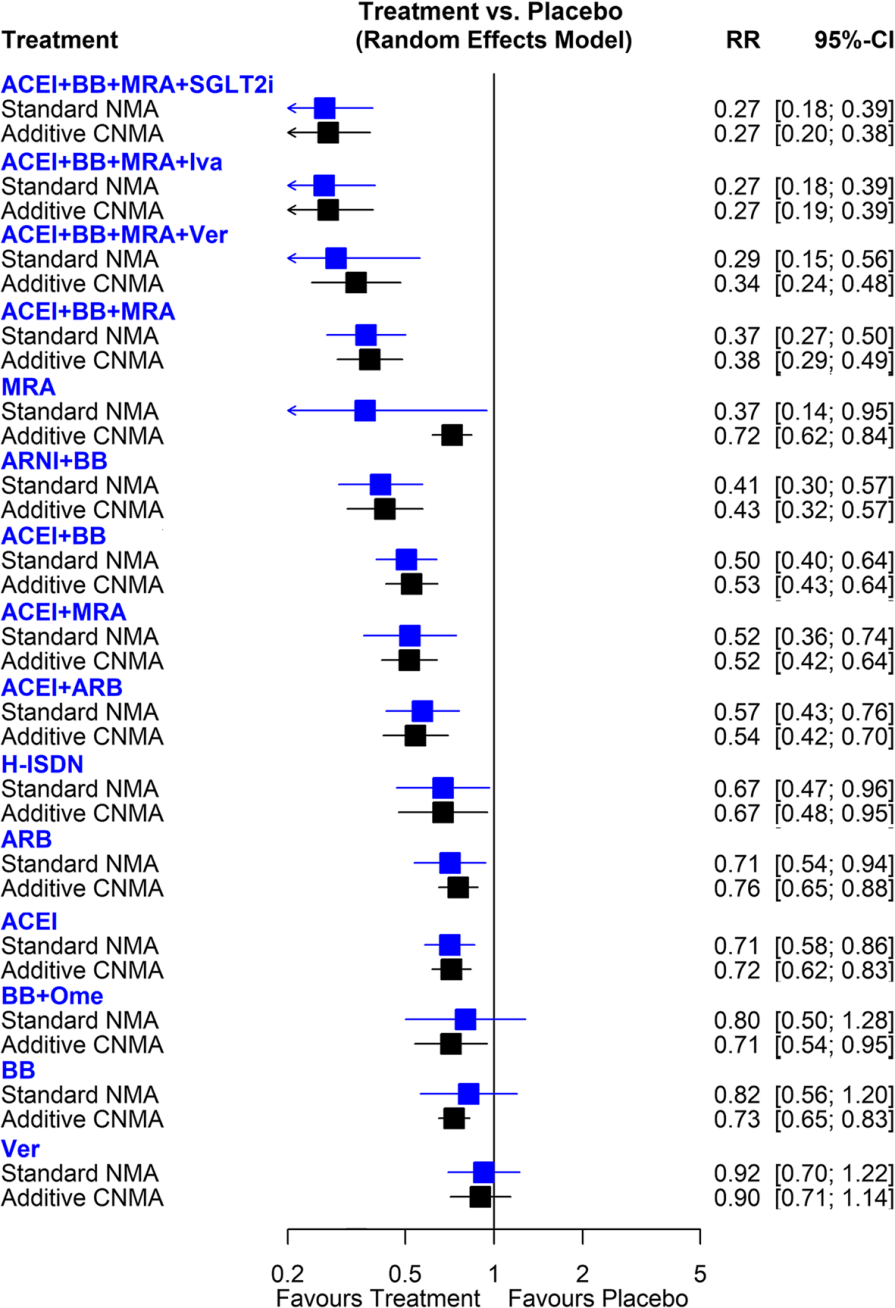
Forest plots of frequentist standard network meta-analysis (NMA) and additive component NMA (CNMA) of pharmaceutical treatments for risk of hospitalization for heart failure in patients with HFrEF. RR, risk ratio; CI, confidence interval; SGLT2i, sodium-glucose co-transporter-2 inhibitors; H-ISDN, Hydralazine and Isosorbide Dinitrate; ACEI, angiotensin II converting enzyme inhibitors; ARB, angiotensin II receptor blockers; ARNI, angiotensin II receptor-neprilysin inhibitors; BB, beta blocker; MRA, mineralocorticoid receptor antagonists; Iva, Ivabradine; Ver, Vericiguat; Ome, Omecamtiv

Figure 2 shows the network of eligible comparisons for HHF, involving 37 RCTs. The standard NMA results for HHF are presented in Fig. 5 and Table S4. Compared with placebo, most treatments, such as ACEI + BB + MRA + SGLT2i (RR, 0.27; 95%CI, 0.18–0.39), ACEI + BB + MRA + Ivabradine (RR, 0.27; 95%CI, 0.18–0.39), ACEI + BB + MRA + Vericiguat (RR, 0.29; 95%CI, 0.15 – 0.56) were significantly associated with a lower risk of HHF. The p-score indicated that ACEI + BB + MRA + SGLT2i (p-score = 0.92) and ACEI + BB + MRA + Ivabradine (p-score = 0.92) were ranked as the best approaches, followed byACEI + BB + MRA + Vericiguat (p-score = 0.86) (Table S5). The point estimates were similar in both standard and additive network meta-analyses (Fig. 5). Because of the increased number of available treatment arms and potential interaction effects in the combination therapies in additive CNMA, several interventions had a narrower CI, resulting in a significant effect, such as BB + Omecamtiv (RR, 0.71; 95%CI, 0.54–0.95) and BB (RR, 0.73; 95%CI, 0.65–0.83). The effects of individual components on all-cause death risk are present in Table S6. ARNI, ACEI, ARB, BB, H-ISDN, Ivabradine, MRA, and SGLT2i were associated with a lower risk of all-cause death.

When checking the consistency in the network, direct and indirect drug comparisons were generally consistent except for the comparison of BB vs. placebo (*p* = 0.01). The heterogeneity was moderate in the network meta-analysis, with I^2^ = 53.6% (95%CI, 27.0%- 70.5%).

### Sensitivity analyses

The results were consistent among the participants with an LVEF ≤ 40%(Figure S2). ACEI + BB + MRA + SGLT2i were proven to be the most effective in reducing all-cause death (RR,0.51; 95%CI, 0.38–0.67), CV death (RR,0.46; 95%CI, 0.33–0.66), and HHF (RR,0.27; 95%CI, 0.18–0.39), followed by ACEI + BB + MRA + Ivabradine, and ARNI + BB or ACEI + BB + MRA (for HHF specifically).

## Discussion

### Main findings

This network meta-analysis of 49 trials involving 90,529 participants with HFrEF, provides further evidence regarding the choice of pharmaceutical treatments for HFrEF. ACEI + BB + MRA + SGLT2i was found to be the most effective treatment for reducing the risk of all-cause death, followed by ACEI + BB + MRA + Vericiguat and ACEI + BB + MRA + Ivabradine. ACEI + BB + MRA + Vericiguat was also found to be the best approach for reducing CV death, followed by ACEI + BB + MRA + SGLT2i and ACEI + BB + MRA + Ivabradine. The best approach for reducing HHF was found to be ACEI + BB + MRA + SGLT2i and ACEI + BB + MRA + Ivabradine, followed by ACEI + BB + MRA + Vericiguat. The results were similar between standard NMA and additive CNMA. These results suggest that combination therapies, particularly those incorporating newer medication, such as SGLT2i, offer the most promising outcomes in terms of reducing mortality and hospitalization risks. The results from additive CNMA also revealed significant benefits for ACEI (or ARNI), BB, and MRA across the outcomes of death and HHF.

### Comparisons with similar studies

Our findings align with previous meta-analyses [69, 70], demonstrating that newer HFrEF medications (such as SGLT2i), enhance the effectiveness of standard treatments in reducing death and improving outcomes. Various combinations of SGLT2i, vericiguat, ivabradine, ACEI, ARNI, BB, and MRA showed superior rates of reducing all-cause death, CV death, and HHF. One network meta-analysis analyzing pharmacological combination treatments in HFrEF patients concluded that the most effective treatment for reducing all-cause death was a combination of ARNI, BB, MRA, and SGLT2i followed by ARNI, BB, MRA, and vericiguat; and ARNI, BB, and MRA [69, 70]. Also, there was a significant benefit of combination therapy in extending the number of life-years by as much as 5 years in patients with HFrEF [69, 70]. Another meta-analysis assessing medication therapy combinations in HFrEF patients found that a combination of ARNI, BB, or MRA, a combination of ACEI, BB, MRA, or SGLT2i, a combination of ACEI, BB, MRA, or Ivabradine were the first three options for reducing mortality and HHF [70]. This study utilized a stepwise induction of pharmacotherapy with SGLT2i + BB + ARNI + MRA being most effective at reducing CV death, hospitalizations, and all-cause death [70]. Our study corroborates these findings, supporting the introduction of combination therapy as the preferred approach for HFrEF patients.

### Clinical implications

Our findings support a stepwise approach to HFrEF treatment, building upon the foundation of ACEI (or ARNI), BB, and MRA. The addition of newer agents such as SGLT2i, vericiguat, or ivabradine to this core regimen appears to offer incremental benefits in reducing death and hospitalization risks. This aligns with the evolving concept of "quadruple therapy" in HFrEF management, where four distinct medication classes are combined to optimize outcomes [71].

The superior performance of combination therapies over monotherapies reinforces the potential benefits of comprehensive pharmacological management in HFrEF. However, it also raises questions about the optimal sequencing of therapies and the potential risks of polypharmacy. For example, risks such as dehydration and kidney failure may be pronounced in patients with polypharmacy [72]. However, this meta-analysis of RCTs did not assess such risks, which limited the applicability of our findings to clinical practice. The populations included in RCT often differ from real-world patients in terms of age, comorbidities, and the intensity of monitoring. Therefore, clinicians should carefully balance the potential benefits of combination therapies against the risks of polypharmacy, considering individual patient characteristics, such as age, comorbidities, and frailty. Future research should focus on real-world effectiveness and safety studies of combination therapies, strategies to mitigate polypharmacy risks, and personalized treatment approaches.

### Strengths and limitations

Our study builds upon and extends previous meta-analyses [69, 70], in several important ways. First, by including the most recent clinical trials, this study provided the most up-to-date evidence on pharmaceutical treatments for HFrEF, reflecting current treatment practices. Second, the use of additive CNMA allowed for a more nuanced evaluation of individual drug components and their contributions to treatment efficacy. Also, the consistency in results between additive CNMA and standard NMA strengthens the reliability of our findings. Third, our comprehensive inclusion of a wide range of pharmacological treatments, from established therapies to newer drug classes, provides a more complete picture of the current HFrEF treatment landscape. However, our results should be interpreted with caution considering some limitations. First, the NMA model assumes consistency between direct and indirect evidence. In our analysis, we found inconsistencies between direct and indirect evidence for some comparisons, which may affect the reliability of our results. Additionally, the additive component model assumes that the effects of individual drugs are additive. This assumption may not hold true in all cases, particularly given the complexity of pharmaceutical therapies for HFrEF. Several factors such as drug interactions, overlapping mechanisms, and dose-dependent effects, may challenge this assumption. Also, the earlier trials primarily compared single agents (e.g., BB or ACEI) with placebo, but the later RCTs have been conducted on a background of established therapies, often including combinations of ACEI, BB, MRA, and newer drug classes such as SGLT2i. This evolution in trial design reflects changes in the standard of care but also introduces challenges in comparing treatments across different eras and may impact the validity of the additive component model. Second, the included studies had differences in population and methodologies. Although we included the population with HFrEF, we still could not guarantee the homogeneity of study populations. The patient populations and study designs varied among the included trials, which could affect the generalizability of the results and introduce heterogeneity in the NMA. Also, there was inconsistency in treatment regimens (e.g., different treatment regimens in included trials), which could affect the interpretation of the results and the validity of the meta-analysis. Third, the absence of patient-level data precludes adjustments of important variables that might have contributed to sources of heterogeneity between trials. For example, the exact information on background therapy was unknown, thus we can not estimate their true treatment effects. In this study, we assumed the participants in one study had background therapy if 60% of participants took the drugs. Also, the absence of patient-level data precluded the ability to conduct important subgroup analyses, e.g., women vs men and older vs. younger patients. Fourth, as the background therapy was not assigned randomly, the therapeutic impacts of a background therapy (e.g., BB) in addition to the randomly assigned drugs (e.g., ACEI) might not correspond to the overall effect of the combined treatment (e.g., ACEI + BB). Fifth, the limited number of trials for some treatments included in the NMA could affect the precision of the estimates and limit the ability to draw conclusions about their efficacy and safety. Finally, there may be potential publication bias in this study. Despite efforts to identify all relevant trials, there is still a possibility of publication bias, where studies with positive results are more likely to be published than those with negative or null results.

## Conclusions

In conclusion, our network meta-analysis provides robust evidence supporting the use of combination therapies in HFrEF management, with newer agents offering incremental benefits when added to established guideline-directed medical therapy. The superior efficacy of ACEI + BB + MRA + SGLT2i in reducing all-cause mortality, along with the promising results for combinations involving vericiguat and ivabradine, suggests that a more comprehensive pharmacological approach may lead to improved outcomes in patients with HFrEF. These results provide a valuable evidence base for informing personalized treatment decisions in HFrEF management. Future research should focus on long-term outcomes, patient subgroups, and potential synergistic effects between different drug combinations to further optimize HFrEF treatment strategies.

## Supplementary Information

Supplementary Material 1.

## Data Availability

All data generated or analysed during this study are included in this published article and its supplementary information files.
