# Supplementary material for: The most effective combination of pharmacological therapy for heart failure with reduced ejection fraction: a network meta-analysis of randomized controlled trials
Source: BMC Cardiovasc Disord. 2024 Nov 23;24:666. doi: 10.1186/s12872-024-04339-3 (PMC11585106; doi:10.1186/s12872-024-04339-3)

**Supplementary materials**

**File S1. Search strategy (Date of search: Feb 22, 2022)**

**Ovid Medline**

| **#** | **Query** |
| --- | --- |
| 1 | exp Heart Failure/ |
| 2 | Cardiomyopathy, Dilated/ |
| 3 | (heart failure or cardiac failure or cardiac insufficiency or cardiomyopath$).tw. |
| 4 | ((cardi$ or myocard$) adj2 (failure$ or insufficien$)).tw. |
| 5 | or/1-4 |
| 6 | exp angiotensin receptor-neprilysin inhibitor/ or ARNI.mp. |
| 7 | (((((LCZ696 or LCZ 696 or LCZ-696 or valsartan) adj6 sacubitril) or valsartan) adj6 sacubitril) or valsartan sacubitril or valsartansacubitril or sacubitril adj6valsartan or sacubitril valsartan or sacubitril-valsartan).mp. |
| 8 | exp dipeptidyl carboxypeptidase inhibitor/ or exp Angiotensin-Converting Enzyme Inhibitors/ |
| 9 | (angiotensin converting enzyme inhibitor or ACEI or ACEI or antagonist$ or inhibitor$ benazepril or captopril or enalapril or fosinopril or imidapril or lisinopril or moexipril or perindopril or quinapril or ramipril or trandolapril or zofenopril or alacepril or cilazapril or spirapril or delapril).mp. |
| 10 | exp beta adrenergic receptor blocking agent/ or exp Adrenergic beta-Antagonists/ |
| 11 | (beta blocker$ or BB or acebutolol or atenolol or betaxolol or bisoprolol or carvedilol or labetalol or metoprolol or nadolol or nebivolol or penbutolol or pindolol or propranolol or sotalol or timolol).mp. |
| 12 | exp aldosterone antagonist/ |
| 13 | (aldosterone antagonist$ or mineralocorticoid-receptor antagonist or MRA or eplerenone or spironolactone or antisterone or aldactone).mp. |
| 14 | exp angiotensin receptor antagonist/ |
| 15 | (angiotensin receptor blocker$ or angiotensin receptor antagonist$ or ARB or azilsartan or candesartan or eprosartan or irbesartan or losartan or olmesartan or telmisartan or valsartan).mp. |
| 16 | (((((sodium-glucose co-transporter 2 or SGLT2 or SGLT2 inhibitor* or sodium glucose) adj6 inhibitor*) or SGLT2 inhibitor* or sodium-glucose) adj6 inhibitor*) or Sodium-Glucose Transporter 2 or sodium glucose-cotransporter 2 or sodium-glucose cotransporter$ or sodium glucose-cotransporter$).mp. |
| 17 | (dapagliflozin or empagliflozin or canagliflozin).mp. |
| 18 | exp ivabradine plus metoprolol/ or exp ivabradine/ or exp carvedilol plus ivabradine/ |
| 19 | (Omecamtiv mecarbil or CK-1827452 or Omecamtiv or mecarbil).mp. |
| 20 | (Vericiguat or guanylate cyclase stimulator or soluble guanylate cyclase stimulator).mp. |
| 21 | (((((Hydralazine-Isosorbide Dinitrate or Hydralazine-Isosorbide) adj6 Dinitrate) or Hydralazine Isosorbide Dinitrate or Hydralazine) adj6 Isosorbide adj6 Dinitrate) or vasodilators).mp. |
| 22 | digoxin.mp. or Digoxin/ |
| 23 | exp phosphodiesterase-5 inhibitor/ |
| 24 | (sidenafil or tadalafil).mp. |
| 25 | (diuretic or loop diuretic).mp. |
| 26 | (furosemide or bumetanide or torasemide or azosemide or eplerenone or hydroclorotiazides).mp. |
| 27 | or/6-26 |
| 28 | "randomized controlled trial".pt. |
| 29 | (random$ or placebo$ or single blind$ or double blind$ or triple blind$).ti,ab. |
| 30 | (retraction of publication or retracted publication).pt. |
| 31 | or/28-30 |
| 32 | (animals not humans).sh. |
| 33 | ((comment or editorial or meta-analysis or practice-guideline or review or letter or journal correspondence) not "randomized controlled trial").pt. |
| 34 | (random sampl$ or random digit$ or random effect$ or random survey or random regression).ti,ab. not "randomized controlled trial".pt. |
| 35 | 32 or 33 or 34 |
| 36 | 31 not 35 |
| 37 | (random$ or placebo$ or single blind$ or double blind$ or triple blind$).ti,ab. |
| 38 | RETRACTED ARTICLE/ |
| 39 | or/37-38 |
| 40 | (animal$ not human$).sh,hw. |
| 41 | (book or conference paper or editorial or letter or review).pt. not exp randomized controlled trial/ |
| 42 | (random sampl$ or random digit$ or random effect$ or random survey or random regression).ti,ab. not exp randomized controlled trial/ |
| 43 | or/40-42 |
| 44 | 39 not 43 |
| 45 | 36 or 44 |
| 46 | 5 and 27 and 45 |

**Embase**

| **#** | **Query** |
| --- | --- |
| 1 | ‘Heart Failure’/exp |
| 3 | (‘heart failure’ or ‘cardiac failure’ or ‘cardiac insufficiency’ or ‘cardiomyopath*’):ab,ti,kw |
| 4 | ((cardi* or myocard*) NEAR/2 (failure* or insufficien*)):ab,ti,kw |
| 5 | or/1-4 |
| 6 | ‘angiotensin receptor-neprilysin inhibitor’/exp or ARNI:ab,ti,kw |
| 7 | (‘LCZ696’ OR ‘LCZ 696’ OR ‘LCZ-696’ OR ‘valsartan NEAR/6 sacubitril’ OR ‘valsartan NEAR/6 sacubitril’ OR ‘valsartan sacubitril’ OR ‘valsartansacubitril’ OR ‘sacubitril NEAR/6 valsartan’ OR ‘sacubitril valsartan’ OR ‘sacubitril-valsartan’):ab,ti,kw |
| 8 | ‘dipeptidyl carboxypeptidase inhibitor’/exp or ‘Angiotensin-Converting Enzyme Inhibitors’/exp |
| 9 | (‘angiotensin converting enzyme inhibitor’ or ACEI or ACEI or benazepril or captopril or enalapril or fosinopril or imidapril or lisinopril or moexipril or perindopril or quinapril or ramipril or trandolapril or zofenopril or alacepril or cilazapril or spirapril or delapril):ab,ti,kw |
| 10 | ‘beta adrenergic receptor blocking agent’/exp |
| 11 | (beta blocker* or BB or acebutolol or atenolol or betaxolol or bisoprolol or carvedilol or labetalol or metoprolol or nadolol or nebivolol or penbutolol or pindolol or propranolol or sotalol or timolol):ab,ti,kw |
| 12 | ‘aldosterone antagonist’/exp |
| 13 | (‘aldosterone antagonist*’ or ‘mineralocorticoid-receptor antagonist’ or MRA or eplerenone or spironolactone or antisterone or aldactone):ab,ti,kw |
| 14 | ‘angiotensin receptor antagonist’/exp |
| 15 | (‘angiotensin receptor blocker*’ or ‘angiotensin receptor antagonist*’ or ARB or azilsartan or candesartan or eprosartan or irbesartan or losartan or olmesartan or telmisartan or valsartan):ab,ti,kw |
| 16 | ‘ivabradine plus metoprolol’/exp or ivabradine/exp or ‘carvedilol plus ivabradine’/exp |
| 17 | (‘Omecamtiv mecarbil’ OR ‘CK-1827452’ OR Omecamtiv OR mecarbil):ab,ti,kw |
| 18 | (Vericiguat OR ‘guanylate cyclase stimulator’ OR ‘soluble guanylate cyclase stimulator’):ab,ti,kw |
| 19 | (‘Hydralazine-Isosorbide Dinitrate’ OR ‘Hydralazine-Isosorbide NEAR/6 Dinitrate’ OR ‘Hydralazine Isosorbide Dinitrate’ OR ‘Hydralazine NEAR/6 Isosorbide NEAR/6 Dinitrate’ OR vasodilators):ab,ti,kw |
| 20 | Digoxin/exp or Digoxin:ab,ti,kw |
| 21 | ‘phosphodiesterase-5 inhibitor’/exp OR (sidenafil or tadalafil):ab,ti,kw |
| 22 | ‘sodium glucose cotransporter 2 inhibitor’/exp |
| 23 | (‘sodium-glucose co-transporter 2’ OR SGLT2 OR ‘SGLT2 inhibitor*’ OR ‘sodium glucose NEAR/6 inhibitor*’ OR ‘sodium-glucose NEAR/6 inhibitor*’ OR ‘Sodium-Glucose Transporter 2’ OR ‘sodium glucose-cotransporter 2’):ab,ti,kw |
| 24 | (dapagliflozin or empagliflozin or canagliflozin):ab,ti,kw |
| 25 | (‘diuretic agent’ OR ‘loop diuretic agent’)/exp |
| 26 | (furosemide or bumetanide or torasemide or azosemide or eplerenone or hydroclorotiazides):ab,ti,kw |
| 27 | or/6-26 |
| 28 | 27 AND [randomized controlled trial]/lim |
| 29 | 28 AND [English]/lim |
| 30 | 29 AND [human]/lim |

**Cochrane Central Register of Controlled Trials**

| # | Query |
| --- | --- |
| 1 | (“heart failure with preserved ejection fraction”[Mesh]) OR (“diastolic heart failure”[Mesh] ) OR (“heart failure with preserved ejection fraction”[Title/Abstract] ) OR (“diastolic heart failure”[Title/Abstract]) |
| 2 | (“randomized controlled trial”[Publication Type] OR (randomized[Publication Typet]) OR “randomized controlled trial”[Title/Abstract] OR randomization [Title/Abstract] |
| 3 | (“angiotensin receptor neprilysin inhibitor”[Mesh]) OR (“sacubitril–valsarta” [Mesh] ) OR (“LCZ696” [Mesh]) OR (“angiotensin receptor neprilysin inhibitor” [Title/Abstract]) OR (“sacubitril–valsarta”[Title/Abstract]) OR (“LCZ696” [Title/Abstract] ) |
| 4 | (“angiotensin converting enzyme inhibitor”[Mesh]) OR (“perindopril” [Mesh] ) OR (“quinapril” [Mesh] ) OR (“perindopri” [Mesh] ) OR (“benazepril” [Mesh] ) OR (“catopril” [Mesh] ) OR (“fosinopril” [Mesh] ) OR (“enalapril” [Mesh] ) OR (“ramipril” [Mesh] ) OR (“angiotensin converting enzyme inhibitor” [Title/Abstract]) OR (“perindopril” [Title/Abstract]) OR (“quinapril” [Title/Abstract] ) OR (“perindopri” [Title/Abstract] ) OR (“benazepril” [Title/Abstract] ) OR (“catopril” [Title/Abstract] ) OR (“Fosinopril” [Title/Abstract] ) OR (“Enalapril” [Title/Abstract] ) OR (“ramipril” [Title/Abstract] ) |
| 5 | (“angiotensin receptor blockers”[Mesh]) OR (“irbesartan” [Mesh] ) OR (“valsartan” [Mesh] ) OR (“candesartan” [Mesh]) OR (“losartan” [Mesh]) OR (“angiotensin receptor blockers”[Title/Abstract]) OR (“Irbesartan” [Title/Abstract] ) OR (“valsartan” [Title/Abstract]) OR (“candesartan” [Title/Abstract]) OR (“losartan” [Title/Abstract] ) |
| 6 | (“beta blockers”[Mesh]) OR (“propranolol” [Mesh] ) OR (“carvedilol” [Mesh] ) OR (“metoprolol” [Mesh]) OR (“bisoprolol” [Mesh]) OR (“beta blockers”[Title/Abstract]) OR (“propranolol”[Title/Abstract]) OR (“carvedilol”[Title/Abstract]) OR (“metoprolol” [Title/Abstract] ) OR (“bisoprolol” [Title/Abstract] ) |
| 7 | (“mineralocorticoid receptor antagonists”[Mesh]) OR (“spirolactone” [Mesh] ) OR (“eplerenone” [Mesh] ) OR (“antisterone” [Mesh]) OR (“aldactone” [Mesh]) OR “mineralocorticoid receptor antagonists”[Title/Abstract]) OR (“spirolactone” [Title/Abstract]) OR (“eplerenone”[Title/Abstract]) OR (“antisterone” [Title/Abstract] ) OR (“aldactone” [Title/Abstract] ) |
| 8 | (“digoxin”[Mesh]) OR (“digoxin” [Title/Abstract] ) |
| 9 | (“phosphodiesterase-5 inhibition”[Mesh]) OR (“sidenafi” [Mesh]) OR (“tadalafil” [Mesh]) OR (“phosphodiesterase-5 inhibition”[Title/Abstract]) OR (“sidenafi” [Title/Abstract]) OR (“tadalafil” [Title/Abstract] ) |
| 10 | (“soluble guanylate cyclase stimulator”[Mesh]) OR (“vericiguat” [Mesh]) OR (“soluble guanylate cyclase stimulator”[Title/Abstract]) OR (“vericiguat” [Title/Abstract]) |
| 11 | (“sodium-glucose cotransporter-2”[Mesh]) OR (“dapagliflozin”[Mesh]) OR (“ canagliflozin” [Mesh] ) OR (“sodium-glucose cotransporter-2”[Title/Abstract]) OR (“dapagliflozin” [Title/Abstract] ) OR (“ canagliflozin” [Title/Abstract] ) |
| 12 | OR/3-11 |
| 13 | 1 AND 2 AND 12 |

**Table S1.** The risk of bias of each trial

| Study | Random sequence generation | Allocation concealment | Blinding (participants and personnel) | Blinding (outcome assessment) | Selective reporting | Incomplete outcome data | Overall |
| --- | --- | --- | --- | --- | --- | --- | --- |
| The CAPRICORN Investigators | Low | Low | Low | Low | Low | Low | Low |
| McMurray 2003 | low | low | low | low | low | low | Low |
| CIBIS-II Investigators and Committees 1999 | high | low | low | low | low | low | High |
| Packer 2001 | Low | Low | Low | Low | Low | Low | Low |
| McMurray 2019 | Low | Low | Low | Low | Low | Low | Low |
| Pitt 1997 | Low | Low | Low | Low | Low | Low | Low |
| Pitt 2000 | Low | Low | Low | Low | Low | Low | Low |
| Packer 2020 | Low | Low | Low | Low | Low | Low | Low |
| Zannad 2011 | Low | Low | Low | Low | Low | low | Low |
| Pitt 2003 | Low | Low | Low | Low | Low | Low | Low |
| Fonarow 1992 | unclear | unclear | high | low | low | low | High |
| Tsutsui 2018 | Low | Low | Low | Low | Low | Low | Low |
| Tsutsui 2019 | Low | Low | Low | Low | Low | Low | Low |
| MERIT-HF | Low | Low | Low | Low | Low | Low | Low |
| He 2015 | unclear | unclear | high | unclear | Low | Low | High |
| Granger 2000 | unclear | unclear | low | low | low | low | unclear |
| McMurray 2014 | low | low | low | low | low | low | low |
| Velazquez 2019 | unclear | unclear | unclear | low | low | low | unclear |
| Packer 1996a | Low | Low | Low | Low | Low | Low | Low |
| Sturm 2000 | Low | Low | Low | Low | Low | Low | Low |
| CIBIS I 1994 | low | low | low | low | low | low | low |
| Erhardt 1995 | unclear | unclear | Low | Low | Low | Low | unclear |
| Teerlink 2021 | low | low | low | low | low | low | low |
| Packer 1996b | low | low | low | low | low | low | low |
| Pitt 1999 | low | low | low | low | low | low | low |
| Dunselman 2001 | low | low | low | low | low | low | low |
| Veldhuisen 1998 | low | low | low | low | low | low | low |
| Armstrong 2020 | low | low | low | low | low | low | low |
| Vizzardi 2014 | low | low | low | low | low | low | low |
| Cohn 1997 | unclear | unclear | unclear | unclear | low | low | unclear |
| Colucci 1996 | unclear | unclear | low | unclear | low | low | unclear |
| Bristow 1996 | low | low | low | low | low | low | low |
| Hori 2004 | low | low | low | low | low | low | low |
| Taylor 2004 | Low | Low | Low | Low | Low | low | Low |
| Boccanelli 2009 | Low | Low | Low | Low | Low | low | Low |
| Beller 1995 | unclear | Low | Low | Low | Low | Low | unclear |
| BEST 2001 | Low | Low | Low | Low | Low | low | Low |
| Brown 1995 | unclear | unclear | Low | Low | Low | low | unclear |
| Captopril-Digoxin group 1988 | unclear | unclear | Low | Low | Low | low | unclear |
| Komajda 2004 | Low | Low | Low | Low | Low | low | Low |
| Widimský 1995 | unclear | unclear | Low | Low | Low | low | unclear |
| Witchitz 2000 | unclear | unclear | Low | Low | Low | low | unclear |
| RESOLVD 2000 | Low | Low | Low | Low | unclear | Low | Low |
| Swedberg 2010 | Low | Low | Low | Low | Low | Low | Low |
| Gheorghiade 2015 | Low | Low | Low | Low | Low | Low | Low |
| SOLVD-prevent 1992 | unclear | unclear | Low | Low | Low | Low | unclear |
| SOLVD-treat 1991 | Low | unclear | Low | Low | Low | Low | unclear |
| Riegger 1999 | unclear | unclear | Low | Low | unclear | Low | unclear |
| Cohn 2001 | Low | Low | Low | Low | Low | Low | Low |

**Table S2.** League table of standard network meta-analysis of the effects of pharmaceutical treatments on all-cause death. Comparisons should be read from up to right in the lower-left corner or from down to left in the upper-right corner.

| ACEI+BB | . | . | . | 1.13 (0.93; 1.38) | 0.71 (0.63; 0.81) | . | 1.17 (0.89; 1.53) | . | . | . | 1.00 (0.47; 2.13) | . | . | . | . |
| --- | --- | --- | --- | --- | --- | --- | --- | --- | --- | --- | --- | --- | --- | --- | --- |
| 0.75 (0.60; 0.94) | ACEI+ARB | . | . | . | 0.96 (0.79; 1.16) | . | . | . | . | . | . | . | . | . | . |
| 1.28 (0.96; 1.71) | 1.71 (1.18; 2.47) | ACEI+BB+MRA  +SGLT2i | . | 0.89 (0.72; 1.09) | . | . | . | . | . | . | . | . | . | . | . |
| 0.72 (0.55; 0.94) | 0.96 (0.71; 1.31) | 0.56 (0.38; 0.83) | ARB | . | 1.00 (0.78; 1.28) | . | . | . | . | . | 0.73 (0.37; 1.44) | . | . | . | 1.47 (0.47; 4.65) |
| 1.13 (0.93; 1.38) | 1.51 (1.12; 2.05) | 0.89 (0.72; 1.09) | 1.57 (1.13; 2.19) | ACEI+BB+MRA | . | 1.08 (0.83; 1.41) | . | . | . | . | . | . | . | 1.32 (0.52; 3.34) | . |
| 0.72 (0.63; 0.81) | 0.96 (0.79; 1.16) | 0.56 (0.41; 0.76) | 0.99 (0.78; 1.26) | 0.63 (0.50; 0.80) | ACEI | . | 1.50 (0.65; 3.43) | . | 1.33 (1.00; 1.76) | . | 0.80 (0.48; 1.33) | 0.52 (0.27; 1.02) | . | . | 0.88 (0.73; 1.06) |
| 1.23 (0.88; 1.70) | 1.64 (1.10; 2.45) | 0.96 (0.68; 1.34) | 1.70 (1.11; 2.60) | 1.08 (0.83; 1.41) | 1.71 (1.20; 2.43) | ACEI+BB+MRA  +Iva | . | . | . | . | . | . | . | . | . |
| 1.16 (0.90; 1.50) | 1.55 (1.10; 2.17) | 0.91 (0.62; 1.33) | 1.61 (1.11; 2.32) | 1.02 (0.74; 1.41) | 1.62 (1.22; 2.14) | 0.94 (0.62; 1.43) | ARNI+BB | . | . | . | . | . | . | . | . |
| 0.74 (0.46; 1.21) | 0.99 (0.60; 1.66) | 0.58 (0.33; 1.02) | 1.03 (0.62; 1.71) | 0.66 (0.39; 1.11) | 1.04 (0.65; 1.67) | 0.61 (0.34; 1.09) | 0.64 (0.37; 1.11) | BB+Ome | . | . | 1.00 (0.77; 1.30) | . | . | . | . |
| 0.95 (0.70; 1.29) | 1.27 (0.90; 1.79) | 0.74 (0.49; 1.13) | 1.32 (0.91; 1.91) | 0.84 (0.58; 1.21) | 1.33 (1.00; 1.76) | 0.78 (0.50; 1.22) | 0.82 (0.55; 1.22) | 1.28 (0.74; 2.21) | ACEI+MRA | . | . | . | . | . | . |
| 0.62 (0.44; 0.88) | 0.83 (0.56; 1.21) | 0.48 (0.31; 0.76) | 0.86 (0.58; 1.28) | 0.55 (0.37; 0.81) | 0.86 (0.62; 1.20) | 0.50 (0.31; 0.81) | 0.53 (0.35; 0.82) | 0.83 (0.47; 1.45) | 0.65 (0.42; 1.00) | Ver | . | . | . | . | 0.96 (0.73; 1.26) |
| 0.74 (0.50; 1.11) | 0.99 (0.64; 1.54) | 0.58 (0.35; 0.96) | 1.03 (0.67; 1.59) | 0.66 (0.42; 1.03) | 1.04 (0.70; 1.54) | 0.61 (0.36; 1.02) | 0.64 (0.40; 1.03) | 1.00 (0.77; 1.30) | 0.78 (0.48; 1.27) | 1.20 (0.74; 1.97) | BB | . | . | . | 0.51 (0.24; 1.08) |
| 0.70 (0.45; 1.07) | 0.93 (0.59; 1.47) | 0.54 (0.33; 0.91) | 0.97 (0.60; 1.55) | 0.61 (0.38; 0.99) | 0.97 (0.65; 1.47) | 0.57 (0.33; 0.98) | 0.60 (0.37; 0.99) | 0.94 (0.50; 1.73) | 0.73 (0.45; 1.20) | 1.13 (0.69; 1.83) | 0.94 (0.54; 1.63) | H-ISDN | . | . | 0.61 (0.37; 0.99) |
| 1.14 (0.41; 3.16) | 1.53 (0.55; 4.27) | 0.89 (0.31; 2.57) | 1.58 (0.56; 4.46) | 1.01 (0.36; 2.84) | 1.60 (0.58; 4.38) | 0.93 (0.32; 2.71) | 0.99 (0.35; 2.81) | 1.53 (0.51; 4.64) | 1.20 (0.42; 3.43) | 1.85 (0.66; 5.18) | 1.53 (0.52; 4.50) | 1.64 (0.56; 4.79) | MRA | . | 0.52 (0.19; 1.40) |
| 1.49 (0.58; 3.87) | 1.99 (0.75; 5.30) | 1.17 (0.45; 3.03) | 2.07 (0.77; 5.56) | 1.32 (0.52; 3.34) | 2.09 (0.80; 5.44) | 1.22 (0.46; 3.21) | 1.29 (0.48; 3.45) | 2.01 (0.69; 5.82) | 1.57 (0.58; 4.26) | 2.42 (0.88; 6.65) | 2.01 (0.71; 5.63) | 2.14 (0.76; 6.08) | 1.31 (0.33; 5.26) | ACEI+BB+MRA  +Ver | . |
| 0.59 (0.48; 0.73) | 0.79 (0.61; 1.03) | 0.46 (0.32; 0.66) | 0.82 (0.61; 1.10) | 0.52 (0.39; 0.70) | 0.83 (0.69; 0.99) | 0.48 (0.33; 0.72) | 0.51 (0.37; 0.71) | 0.80 (0.49; 1.29) | 0.62 (0.45; 0.87) | 0.96 (0.73; 1.26) | 0.80 (0.53; 1.20) | 0.85 (0.57; 1.27) | 0.52 (0.19; 1.40) | 0.40 (0.15; 1.05) | Placebo |

ACEI, angiotensin II converting enzyme inhibitors; ARB, angiotensin II receptor blockers; ARNI, angiotensin II receptor-neprilysin inhibitors; BB, beta blocker; H-ISDN, Hydralazine and Isosorbide Dinitrate; MRA, mineralocorticoid receptor antagonists; SGLT2i, sodium-glucose co-transporter-2 inhibitors.

**Table S3.** League table of network meta-analysis of the effects of pharmaceutical treatments on cardiovascular death. Comparisons should be read from up to right in the lower-left corner or from down to left in the upper-right corner.

| ACEI+BB | . | . | . | 1.19 (0.99; 1.43) | 0.71 (0.62; 0.82) | . | 1.23 (0.97; 1.57) | . | . | . | 0.69 (0.29; 1.63) | . | . | . | . |
| --- | --- | --- | --- | --- | --- | --- | --- | --- | --- | --- | --- | --- | --- | --- | --- |
| 0.83 (0.62; 1.10) | ACEI+ARB | . | . | . | 0.87 (0.67; 1.12) | . | . | . | . | . | . | . | . | . | . |
| 1.35 (1.03; 1.77) | 1.64 (1.10; 2.43) | ACEI+BB+MRA  +SGLT2i | . | 0.88 (0.72; 1.07) | . | . | . | . | . | . | . | . | . | . | . |
| 0.58 (0.33; 1.01) | 0.70 (0.38; 1.28) | 0.42 (0.23; 0.80) | ARB | . | 1.35 (0.76; 2.40) | . | . | . | . | . | 0.78 (0.36; 1.70) | . | . | . | . |
| 1.19 (0.99; 1.43) | 1.43 (1.02; 2.02) | 0.88 (0.72; 1.07) | 2.06 (1.14; 3.74) | ACEI+BB+MRA | . | 1.09 (0.86; 1.38) | . | . | . | . | . | . | . | 1.58 (0.62; 4.07) | . |
| 0.72 (0.63; 0.82) | 0.87 (0.67; 1.12) | 0.53 (0.39; 0.72) | 1.25 (0.72; 2.17) | 0.60 (0.48; 0.76) | ACEI | . | . | . | 1.36 (1.05; 1.76) | . | 0.89 (0.52; 1.55) | 0.47 (0.24; 0.94) | . | . | 0.88 (0.73; 1.05) |
| 1.29 (0.95; 1.75) | 1.56 (1.03; 2.37) | 0.95 (0.70; 1.30) | 2.24 (1.18; 4.27) | 1.09 (0.86; 1.38) | 1.80 (1.29; 2.51) | ACEI+BB+MRA  +Iva | . | . | . | . | . | . | . | . | . |
| 1.23 (0.97; 1.57) | 1.49 (1.03; 2.17) | 0.91 (0.64; 1.31) | 2.15 (1.16; 3.97) | 1.04 (0.77; 1.41) | 1.72 (1.31; 2.27) | 0.96 (0.65; 1.41) | ARNI+BB | . | . | . | . | . | . | . | . |
| 0.60 (0.34; 1.05) | 0.72 (0.39; 1.33) | 0.44 (0.23; 0.83) | 1.04 (0.52; 2.05) | 0.50 (0.28; 0.91) | 0.83 (0.47; 1.46) | 0.46 (0.24; 0.88) | 0.48 (0.26; 0.90) | BB+Ome | . | . | 1.01 (0.80; 1.28) | . | . | . | . |
| 0.97 (0.73; 1.30) | 1.18 (0.82; 1.69) | 0.72 (0.48; 1.07) | 1.69 (0.92; 3.12) | 0.82 (0.58; 1.16) | 1.36 (1.05; 1.76) | 0.75 (0.50; 1.15) | 0.79 (0.54; 1.15) | 1.63 (0.88; 3.03) | ACEI+MRA | . | . | . | . | . | . |
| 0.67 (0.48; 0.94) | 0.81 (0.54; 1.21) | 0.49 (0.32; 0.76) | 1.17 (0.62; 2.19) | 0.57 (0.39; 0.83) | 0.93 (0.69; 1.27) | 0.52 (0.33; 0.82) | 0.54 (0.36; 0.82) | 1.13 (0.59; 2.13) | 0.69 (0.46; 1.03) | Ver | . | . | . | . | 0.94 (0.73; 1.20) |
| 0.60 (0.36; 1.01) | 0.73 (0.41; 1.29) | 0.44 (0.25; 0.80) | 1.05 (0.55; 1.99) | 0.51 (0.29; 0.88) | 0.84 (0.50; 1.40) | 0.47 (0.26; 0.85) | 0.49 (0.27; 0.86) | 1.01 (0.80; 1.28) | 0.62 (0.35; 1.10) | 0.90 (0.50; 1.63) | BB | . | . | . | . |
| 0.34 (0.17; 0.68) | 0.41 (0.20; 0.85) | 0.25 (0.12; 0.53) | 0.59 (0.24; 1.42) | 0.29 (0.14; 0.59) | 0.47 (0.24; 0.94) | 0.26 (0.12; 0.56) | 0.27 (0.13; 0.57) | 0.57 (0.23; 1.38) | 0.35 (0.17; 0.72) | 0.50 (0.24; 1.07) | 0.56 (0.24; 1.32) | H-ISDN | . | . | . |
| 1.09 (0.36; 3.35) | 1.32 (0.42; 4.14) | 0.81 (0.25; 2.55) | 1.90 (0.55; 6.57) | 0.92 (0.30; 2.87) | 1.52 (0.50; 4.63) | 0.85 (0.26; 2.70) | 0.88 (0.28; 2.78) | 1.83 (0.53; 6.37) | 1.12 (0.36; 3.51) | 1.63 (0.53; 5.02) | 1.81 (0.53; 6.17) | 3.22 (0.87; 11.92) | MRA | . | 0.58 (0.19; 1.73) |
| 1.88 (0.72; 4.91) | 2.27 (0.83; 6.20) | 1.39 (0.53; 3.63) | 3.26 (1.07; 9.96) | 1.58 (0.62; 4.07) | 2.62 (0.99; 6.91) | 1.45 (0.55; 3.85) | 1.52 (0.56; 4.09) | 3.15 (1.03; 9.64) | 1.93 (0.71; 5.26) | 2.80 (1.01; 7.75) | 3.12 (1.04; 9.30) | 5.54 (1.69; 18.20) | 1.72 (0.39; 7.53) | ACEI+BB+MRA  +Ver | . |
| 0.63 (0.50; 0.79) | 0.76 (0.56; 1.04) | 0.46 (0.33; 0.66) | 1.09 (0.61; 1.95) | 0.53 (0.40; 0.71) | 0.88 (0.73; 1.05) | 0.49 (0.33; 0.71) | 0.51 (0.37; 0.71) | 1.06 (0.59; 1.90) | 0.65 (0.47; 0.88) | 0.94 (0.73; 1.20) | 1.04 (0.61; 1.79) | 1.86 (0.91; 3.77) | 0.58 (0.19; 1.73) | 0.34 (0.12; 0.90) | Placebo |

ACEI, angiotensin II converting enzyme inhibitors; ARB, angiotensin II receptor blockers; ARNI, angiotensin II receptor-neprilysin inhibitors; BB, beta blocker; H-ISDN, Hydralazine and Isosorbide Dinitrate; MRA, mineralocorticoid receptor antagonists; SGLT2i, sodium-glucose co-transporter-2 inhibitors.

**Table S4.** League table of network meta-analysis of the effects of pharmaceutical treatments on hospitalization for heart failure. Comparisons should be read from up to right in the lower-left corner or from down to left in the upper-right corner.

| ACEI+BB | . | . | . | 1.37 (1.13; 1.67) | 0.71 (0.62; 0.82) | . | 1.22 (0.92; 1.62) | . | . | . | 0.58 (0.23; 1.51) | . | . | . | . |
| --- | --- | --- | --- | --- | --- | --- | --- | --- | --- | --- | --- | --- | --- | --- | --- |
| 0.88 (0.69; 1.13) | ACEI+ARB | . | . | . | 0.81 (0.66; 1.00) | . | . | . | . | . | . | . | . | . | . |
| 1.89 (1.41; 2.54) | 2.15 (1.46; 3.15) | ACEI+BB+MRA  +SGLT2i | . | 0.72 (0.58; 0.90) | . | . | . | . | . | . | . | . | . | . | . |
| 0.71 (0.55; 0.92) | 0.81 (0.60; 1.09) | 0.38 (0.26; 0.55) | ARB | . | 0.99 (0.79; 1.24) | . | . | . | . | . | 1.01 (0.65; 1.56) | . | . | . | 0.69 (0.32; 1.52) |
| 1.37 (1.13; 1.67) | 1.55 (1.13; 2.13) | 0.72 (0.58; 0.90) | 1.92 (1.40; 2.65) | ACEI+BB+MRA | . | 1.39 (1.08; 1.79) | . | . | . | . | . | . | . | 1.27 (0.71; 2.26) | . |
| 0.71 (0.62; 0.82) | 0.81 (0.66; 1.00) | 0.38 (0.27; 0.52) | 1.00 (0.81; 1.24) | 0.52 (0.41; 0.66) | ACEI | . | 1.74 (1.08; 2.80) | . | 1.36 (1.01; 1.85) | . | 1.00 (0.68; 1.48) | . | . | . | 0.67 (0.55; 0.83) |
| 1.90 (1.38; 2.62) | 2.16 (1.44; 3.24) | 1.01 (0.72; 1.40) | 2.67 (1.78; 4.02) | 1.39 (1.08; 1.79) | 2.67 (1.88; 3.78) | ACEI+BB+MRA  +Iva | . | . | . | . | . | . | . | . | . |
| 1.22 (0.96; 1.57) | 1.39 (0.99; 1.94) | 0.65 (0.44; 0.95) | 1.72 (1.22; 2.42) | 0.89 (0.65; 1.23) | 1.72 (1.32; 2.23) | 0.64 (0.43; 0.96) | ARNI+BB | . | . | . | . | . | . | . | . |
| 0.63 (0.40; 0.99) | 0.72 (0.44; 1.16) | 0.33 (0.19; 0.57) | 0.89 (0.56; 1.39) | 0.46 (0.28; 0.75) | 0.88 (0.57; 1.37) | 0.33 (0.19; 0.58) | 0.52 (0.31; 0.86) | BB+Ome | . | . | 0.98 (0.74; 1.28) | . | . | . | . |
| 0.97 (0.70; 1.36) | 1.10 (0.76; 1.59) | 0.51 (0.33; 0.80) | 1.37 (0.94; 1.98) | 0.71 (0.48; 1.04) | 1.36 (1.01; 1.85) | 0.51 (0.32; 0.81) | 0.79 (0.53; 1.19) | 1.54 (0.91; 2.62) | ACEI+MRA | . | . | . | . | . | . |
| 0.55 (0.38; 0.79) | 0.62 (0.42; 0.92) | 0.29 (0.18; 0.46) | 0.77 (0.52; 1.14) | 0.40 (0.26; 0.60) | 0.77 (0.54; 1.08) | 0.29 (0.18; 0.47) | 0.45 (0.29; 0.69) | 0.87 (0.50; 1.49) | 0.56 (0.36; 0.89) | Ver | . | . | . | . | 0.92 (0.70; 1.22) |
| 0.62 (0.43; 0.88) | 0.70 (0.47; 1.04) | 0.32 (0.20; 0.52) | 0.86 (0.60; 1.24) | 0.45 (0.30; 0.68) | 0.86 (0.61; 1.21) | 0.32 (0.20; 0.52) | 0.50 (0.33; 0.77) | 0.98 (0.74; 1.28) | 0.63 (0.40; 1.00) | 1.13 (0.70; 1.80) | BB | . | . | . | 2.97 (1.06; 8.32) |
| 0.75 (0.49; 1.16) | 0.85 (0.54; 1.35) | 0.40 (0.24; 0.67) | 1.06 (0.67; 1.67) | 0.55 (0.34; 0.88) | 1.05 (0.70; 1.59) | 0.40 (0.23; 0.68) | 0.61 (0.38; 1.00) | 1.19 (0.66; 2.15) | 0.77 (0.46; 1.29) | 1.38 (0.87; 2.17) | 1.22 (0.72; 2.06) | H-ISDN | . | . | 0.67 (0.47; 0.96) |
| 1.38 (0.52; 3.67) | 1.56 (0.58; 4.21) | 0.73 (0.26; 2.02) | 1.94 (0.72; 5.21) | 1.01 (0.37; 2.73) | 1.93 (0.73; 5.10) | 0.72 (0.26; 2.03) | 1.13 (0.41; 3.08) | 2.19 (0.76; 6.30) | 1.42 (0.51; 3.91) | 2.52 (0.94; 6.79) | 2.24 (0.81; 6.23) | 1.83 (0.66; 5.07) | MRA | . | 0.37 (0.14; 0.95) |
| 1.73 (0.94; 3.19) | 1.97 (1.02; 3.80) | 0.92 (0.49; 1.70) | 2.44 (1.26; 4.72) | 1.27 (0.71; 2.26) | 2.43 (1.30; 4.54) | 0.91 (0.48; 1.71) | 1.42 (0.73; 2.74) | 2.75 (1.28; 5.89) | 1.78 (0.89; 3.57) | 3.17 (1.56; 6.47) | 2.82 (1.39; 5.74) | 2.31 (1.09; 4.87) | 1.26 (0.40; 3.99) | ACEI+BB+MRA  +Ver | . |
| 0.50 (0.40; 0.64) | 0.57 (0.43; 0.76) | 0.27 (0.18; 0.39) | 0.71 (0.54; 0.94) | 0.37 (0.27; 0.50) | 0.71 (0.58; 0.86) | 0.27 (0.18; 0.39) | 0.41 (0.30; 0.57) | 0.80 (0.50; 1.28) | 0.52 (0.36; 0.74) | 0.92 (0.70; 1.22) | 0.82 (0.56; 1.20) | 0.67 (0.47; 0.96) | 0.37 (0.14; 0.95) | 0.29 (0.15; 0.56) | Placebo |

ACEI, angiotensin II converting enzyme inhibitors; ARB, angiotensin II receptor blockers; ARNI, angiotensin II receptor-neprilysin inhibitors; BB, beta blocker; H-ISDN, Hydralazine and Isosorbide Dinitrate; MRA, mineralocorticoid receptor antagonists; SGLT2i, sodium-glucose co-transporter-2 inhibitors.

**Table S5.** SUCRA (P-score) showing the probability of pharmaceutical treatment being the most effective one for all-cause mortality, cardiovascular (CV) death, and hospitalization for heart failure (HHF).

| Treatments | **All-cause death** | | **CV death** | | **HHF** | |
| --- | --- | --- | --- | --- | --- | --- |
|  | p-score | rank | p-score | rank | p-score | rank |
| ACEI+BB+MRA+SGLT2i | 0.86 | 1 | 0.87 | 2 | 0.92 | 1 |
| ACEI+BB+MRA+ Vericiguat | 0.83 | 2 | 0.90 | 1 | 0.86 | 3 |
| ACEI+BB+MRA+ Ivabradine | 0.82 | 3 | 0.83 | 3 | 0.92 | 1 |
| ARNI+BB | 0.78 | 4 | 0.79 | 4 | 0.71 | 6 |
| ACEI+BB+MRA | 0.75 | 5 | 0.75 | 5 | 0.76 | 4 |
| MRA | 0.67 | 6 | 0.64 | 6 | 0.72 | 5 |
| ACEI+BB | 0.62 | 7 | 0.60 | 7 | 0.58 | 7 |
| ACEI+MRA | 0.59 | 8 | 0.60 | 8 | 0.55 | 8 |
| ACEI+ARB | 0.34 | 9 | 0.47 | 9 | 0.48 | 9 |
| BB+ Omecamtiv | 0.33 | 10 | 0.23 | 13 | 0.21 | 13 |
| BB | 0.33 | 10 | 0.23 | 12 | 0.18 | 14 |
| ARB | 0.30 | 12 | 0.21 | 15 | 0.30 | 11 |
| ACEI | 0.29 | 13 | 0.35 | 10 | 0.30 | 12 |
| H-ISDN | 0.27 | 14 | 0.03 | 16 | 0.35 | 10 |
| Vericiguat | 0.15 | 15 | 0.29 | 11 | 0.11 | 15 |
| Placebo | 0.08 | 16 | 0.22 | 14 | 0.04 | 16 |

ACEI, angiotensin II converting enzyme inhibitors; ARB, angiotensin II receptor blockers; ARNI, angiotensin II receptor-neprilysin inhibitors; BB, beta blocker; H-ISDN, Hydralazine and Isosorbide Dinitrate; MRA, mineralocorticoid receptor antagonists; SGLT2i, sodium-glucose co-transporter-2 inhibitors

**Table S6.** Effect of individual components on risk of all-cause death, cardiovascular (CV) death, and hospitalization for heart failure (HHF) using Additive componet network meta-analysis. RR, risk ratio

| Treatment | **All-cause deth** | **CV death** | **HHF** |
| --- | --- | --- | --- |
|  | RR[95%CI] | RR[95%CI] | RR[95%CI] |
| ACEI | 0.85 [0.74; 0.98] | 0.84 [0.72; 0.98] | 0.72 [0.62; 0.83] |
| ARB | 0.91 [0.79; 1.06] | 0.88 [0.71; 1.09] | 0.76 [0.65; 0.88] |
| ARNI | 0.74 [0.56; 0.96] | 0.68 [0.52; 0.89] | 0.58 [0.44; 0.77] |
| BB | 0.73 [0.65; 0.81] | 0.73 [0.65; 0.83] | 0.73 [0.65; 0.83] |
| H-ISDN | 0.85 [0.58; 1.26] | 1.79 [0.89; 3.58] | 0.67 [0.48; 0.95] |
| Ivabradine | 0.92 [0.73; 1.17] | 0.92 [0.74; 1.14] | 0.72 [0.55; 0.93] |
| MRA | 0.82 [0.71; 0.95] | 0.80 [0.70; 0.92] | 0.72 [0.62; 0.84] |
| Omecamtiv | 1.00 [0.79; 1.26] | 1.01 [0.82; 1.24] | 0.98 [0.76; 1.26] |
| SGLT2i | 0.89 [0.73; 1.07] | 0.88 [0.73; 1.05] | 0.72 [0.59; 0.89] |
| Vericiguat | 0.94 [0.75; 1.19] | 0.92 [0.74; 1.14] | 0.90 [0.71; 1.14] |

ACEI, angiotensin II converting enzyme inhibitors; ARB, angiotensin II receptor blockers; ARNI, angiotensin II receptor-neprilysin inhibitors; BB, beta blocker; H-ISDN, Hydralazine and Isosorbide Dinitrate; MRA, mineralocorticoid receptor antagonists; SGLT2i, sodium-glucose co-transporter-2 inhibitors.

**Figure S1.** The comparison-adjusted funnel plot and Egger’s test to evaluate the publication bias or small-study effects in terms of all-cause death (A), cardiovascular death (B), and hospitalization for heart failure (HHF) (C). ACEI, angiotensin II converting enzyme inhibitors; ARB, angiotensin II receptor blockers; ARNI, angiotensin II receptor-neprilysin inhibitors; BB, beta blocker; H-ISDN, Hydralazine and Isosorbide Dinitrate; MRA, mineralocorticoid receptor antagonists; SGLT2i, sodium-glucose co-transporter-2 inhibitors


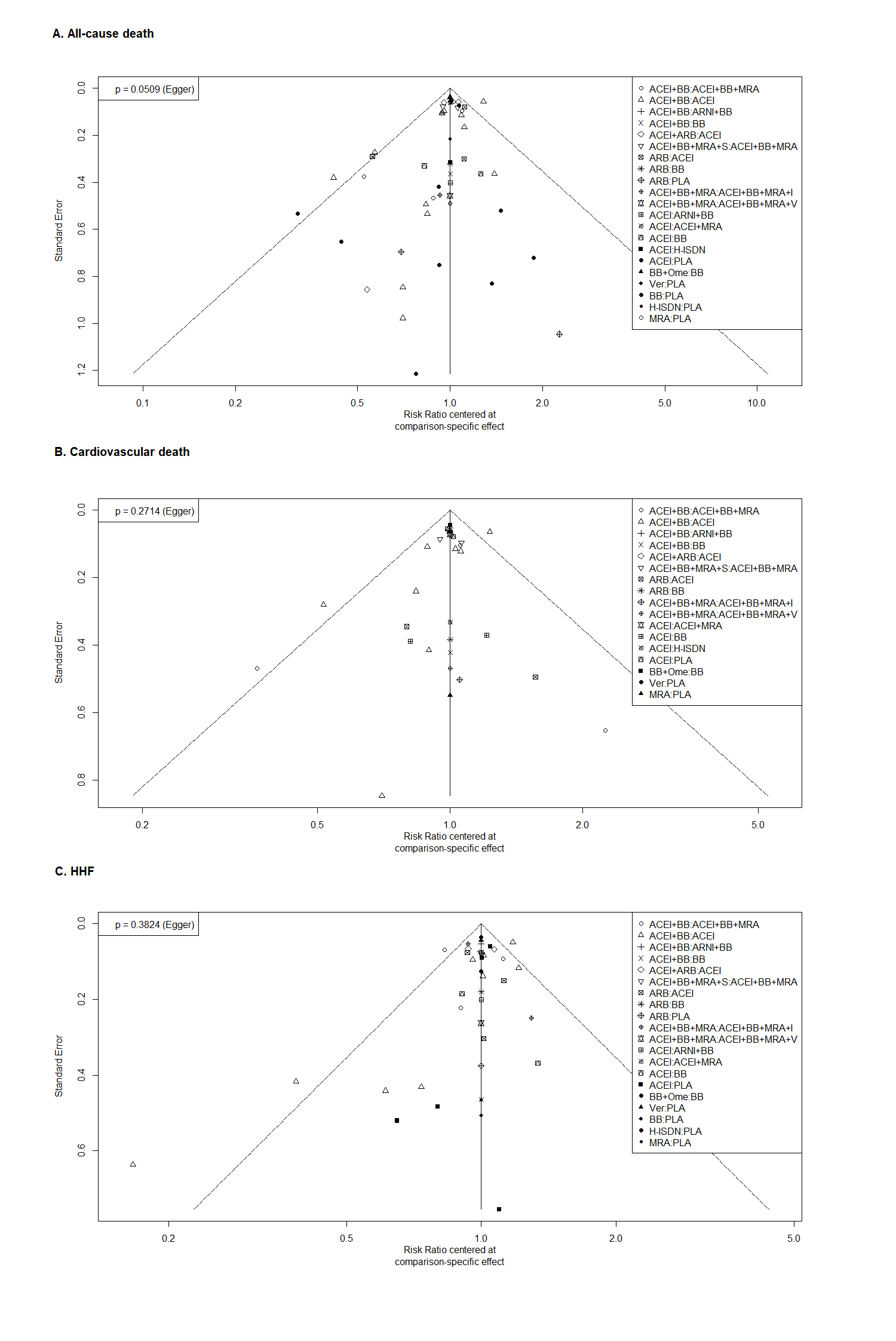


**Figure S2.** Forest plots of frequentist standard network meta-analysis (NMA) and additive component NMA (CNMA) of pharmaceutical treatments for risk of all-cause death (A), cardiovascular death (B), and hospitalization for heart failure (HHF) (C) in patients with HFrEF and a left ventricular ejection fraction ≤ 40%. RR, risk ratio; CI, confidence interval; SGLT2i, sodium-glucose co-transporter-2 inhibitors; H-ISDN, Hydralazine and Isosorbide Dinitrate; ACEI, angiotensin II converting enzyme inhibitors; ARB, angiotensin II receptor blockers; ARNI, angiotensin II receptor-neprilysin inhibitors; BB, beta blocker; MRA, mineralocorticoid receptor antagonists; Iva, Ivabradine; Ver, Vericiguat; Ome, Omecamtiv.


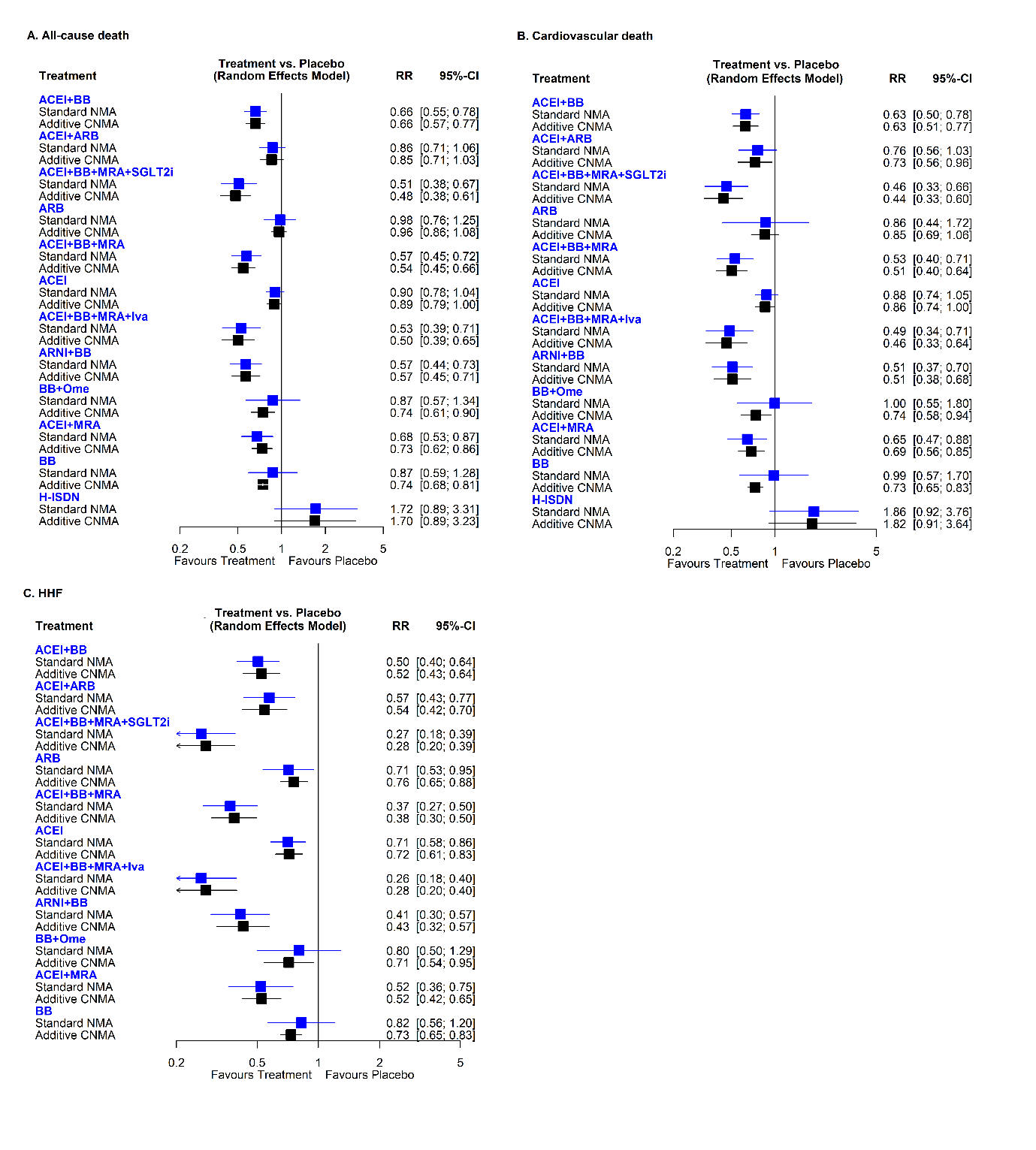

Supplement: Supplementary file 1 — Supplementary Material 1. [file 12872_2024_4339_MOESM1_ESM.docx]
